# Targeting CD300a Signaling With a Phosphatidylserine‐Presenting Hydrogel Rescues Macrophage Dysfunction and Accelerates Diabetic Wound Healing

**DOI:** 10.1002/adhm.71648

**Published:** 2026-08-30

**Authors:** Lele Wu, Xuetong Hao, Yang Zou, Xinglin Chen, Junyan Kong, Chunning Gu, Wenjie Ma, Zhongjun Yang, Huifen Liu, Mengmeng Liu, Xin Tong

**Affiliations:** ^1^ Nanjing Stomatological Hospital, Affiliated Hospital of Medical School, Institute of Stomatology Nanjing University Nanjing China; ^2^ Department of Stomatology Qilu Hospital (Qingdao) Cheeloo College of Medicine Shandong University Qingdao China

**Keywords:** bioactive hydrogel, CD300a, diabetic wound healing, efferocytosis‐independent signaling, macrophage reprogramming, NF‐κB signaling, phosphatidylserine

## Abstract

The delayed healing of diabetic wounds involves both impaired efferocytosis and dysfunctional phosphatidylserine (PS) receptor signaling, leading to macrophage defects in migration, persistent M1 polarization, and diminished cellular resilience. To address this multifaceted pathology, a bioactive wound dressing was developed by incorporating PEGylated RGD‐grafted phosphatidylserine liposomes (PEG/RGD‐PSLs) into a photocrosslinkable hyaluronic acid methacryloyl (HAMA) hydrogel matrix. This platform provides sustained, delivery of a biomimetic “eat‐me” signal while actively reprogramming macrophage behavior. Under diabetic‐mimicking stress conditions, PEG/RGD‐PSLs significantly enhanced macrophage migration, promoted M1‐to‐M2 phenotypic transition, and conferred robust cytoprotection by preserving mitochondrial integrity, attenuating oxidative stress, and suppressing pathological extracellular vesicle release. Mechanistically, PS binding upregulated the inhibitory receptor CD300a, which suppressed the MyD88/NF‐κB pathway and downregulated pro‐inflammatory genes. Critically, siRNA‐mediated CD300a knockdown abolished these anti‐inflammatory and NF‐κB‐suppressive effects, establishing CD300a as necessary for therapeutic action. In a diabetic rat model, a single application of the bioactive hydrogel significantly accelerated wound closure, stimulated angiogenesis, improved organized collagen deposition, and actively shifted the wound immune microenvironment toward a pro‐reparative M2‐dominant state. Collectively, this study identifies the PS/CD300a/NF‐κB axis as a key regulatory node for rescuing macrophage dysfunction and establishes a functionally active hydrogel‐based therapy for chronic diabetic wounds.

## Graphical abstract

*Schematic illustration of the proposed mechanism*: PEG/RGD‐PSLs mimic apoptotic cells to engage PS receptors (notably CD300a), transducing an inhibitory signal that suppresses the MyD88/NF‐κB pathway, leading to global anti‐inflammatory and pro‐reparative effects.

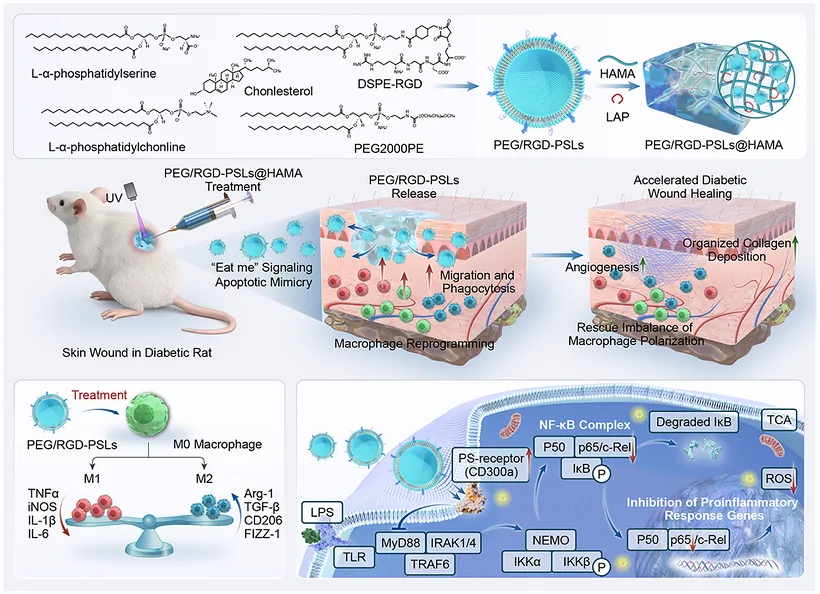

## 1 Introduction

Diabetic wound healing impairment poses a critical clinical challenge, characterized by prolonged treatment durations, substantial healthcare costs, and a heightened risk of complications such as amputation [1]. Normal wound repair progresses through a well‐coordinated sequence of overlapping phases—hemostasis, inflammation, proliferation, and tissue remodeling [2, 3]. In diabetes, persistent hyperglycemia and tissue hypoxia disrupt this orderly progression, leading to chronic inflammation, cellular dysfunction, and failure of wound closure [4, 5, 6]. Current management strategies, including debridement, infection control, and advanced dressings, remain inadequate in addressing these complex pathological mechanisms [5, 7, 8, 9].

Central to this pathology is the multifaceted dysfunction of macrophages, the master regulators of innate immunity, inflammation resolution, and tissue repair [10, 11]. Under physiological conditions, macrophages dynamically transition from a pro‐inflammatory (M1) to a pro‐healing (M2) phenotype to orchestrate effective repair [10]. In the diabetic milieu, however, they exhibit a characteristic triad of impairments: compromised chemotactic migration, locked pro‐inflammatory M1 polarization, and heightened metabolic fragility [4, 12]. Hyperglycemia perpetuates excessive M1 polarization, driving the sustained release of inflammatory cytokines such as IL‐1β, TNF‐α, and IL‐6, which amplifies oxidative stress and delays the transition to the proliferative phase [11]. In contrast, the pro‐reparative (M2) macrophage phenotype facilitates wound healing by secreting anti‐inflammatory cytokines and essential growth factors [11, 13]. Thus, macrophages represent a promising therapeutic target in diabetic wound healing [10, 14].

A key mechanism governing macrophage function and inflammation resolution is efferocytosis—the phagocytic clearance of apoptotic cells. This process stimulates the release of anti‐inflammatory mediators (e.g., TGF‐β, IL‐10) [15, 16] and upregulates reparative genes, thereby facilitating the transition to tissue regeneration [17, 18]. In chronic wounds, efferocytosis is markedly impaired, contributing to persistent neutrophil infiltration, reduced anti‐inflammatory macrophage activity, defective angiogenesis, and elevated protease activity [19, 20]. This dysfunction encompasses defects in “find‐me”/“eat‐me” signaling, phagocytic receptor dysregulation, and disrupted downstream cascades [21, 22]. Consequently, therapeutic strategies that enhance efferocytosis have emerged as promising approaches to restore immune homeostasis and support tissue repair, such as the use of extracellular vesicles and biomimetic nanotherapeutics [23, 24, 25, 26].

Among endogenous “eat‐me” signals, phosphatidylserine (PS)—a phospholipid externalized on apoptotic cell membranes—plays a critical role. PS engages specific receptors on macrophages (e.g., TREM2, BAI1, CD300a) to initiate efferocytosis, suppress inflammation, and promote an M2‐like reparative phenotype [24, 27]. Biomimetic strategies leveraging PS‐presenting liposomes (PSLs) have shown potential in modulating macrophage function and attenuating inflammation [28, 29]. Our previous work demonstrated the therapeutic potential of RGD‐grafted PS‐containing liposomes in attenuating inflammation and accelerating bone tissue regeneration [30]. These findings position PSLs as a promising candidate for diabetic wound therapy. However, their translational potential is limited by rapid clearance, lack of tissue specificity, and poor retention in the dynamic wound environment [31, 32].

To overcome these limitations, we engineered a multi‐functional signaling unit: PEGylated RGD‐grafted phosphatidylserine liposomes (PEG/RGD‐PSLs). This design integrates (1) PS, which provides the core “eat‐me” signal for macrophage engagement; (2) RGD peptide, which confers targeting specificity to αvβ3 integrins overexpressed on activated macrophages [33, 34]; and (3) PEGylation, which prolongs systemic circulation half‐life. To enable localized and sustained delivery, we incorporated PEG/RGD‐PSLs into a photocrosslinkable hyaluronic acid methacryloyl (HAMA) hydrogel. While hydrogels are widely used as moisture‐retentive wound dressings, their passive nature often limits their ability to correct profound cellular dysfunction [35, 36]. This has spurred research into bioactive dressings capable of dynamically modulating the wound microenvironment, with anti‐inflammatory hydrogels being a prime example [5, 35, 37]. We therefore hypothesized that an active hydrogel platform capable of sustained PS/RGD co‐signaling could serve as an instructive wound interface, simultaneously promoting macrophage recruitment, phenotypic reprogramming, and cytoprotection under diabetic stress.

Herein, we report the development and comprehensive evaluation of this bioactive hydrogel system. We demonstrate its ability to reverse diabetic macrophage dysfunction across transcriptional, phenotypic, and ultrastructural levels, and elucidate the underlying mechanism involving the PS/CD300a/NF‐κB axis. Furthermore, we show that a single application of the hydrogel significantly accelerates wound closure and improves healing quality in a diabetic rat model. This work establishes a targeted, mechanism‐driven biomaterial strategy to reprogram the immune microenvironment and restore regenerative capacity in chronic diabetic wounds.

## 2 Results

### 2.1 Fabrication and Characterization of PEG/RGD‐PSLs and the Bioactive Hydrogel

To modulate the immunomodulatory function of PSLs, we engineered a series of PSLs grafted with RGD peptides and varying densities of polyethylene glycol (PEG). A series of liposomes with increasing PEGylation molar ratios (RGD‐PSLs, 0.25%PEG/RGD‐PSLs, 0.5%PEG/RGD‐PSLs, 0.75%PEG/RGD‐PSLs, and 1%PEG/RGD‐PSLs) were successfully synthesized. Dynamic light scattering (DLS) revealed a PEG‐dependent increase in hydrodynamic diameter from 141.8 ± 5.7 nm (RGD‐PSLs) to 192.3 ± 2.3 nm, accompanied by a corresponding decrease in absolute zeta potential from 43.9 ± 1.1 to 16.6 ± 0.4 mV (Figures 1B,D and S1A). Notably, the zeta potential of the 1%PEG/RGD‐PSLs formulation approached the threshold for colloidal instability (typically > |−30| mV), guiding the selection of formulations with ≤ 0.75% PEG for subsequent studies. Successful PEG conjugation was verified by ^1^H‐NMR spectroscopy with the characteristic ethylene oxide proton signal at ∼3.6 ppm (Figures 1C and S1B). Transmission electron microscopy (TEM) confirmed the formation of unilamellar and occasionally multilamellar vesicular structures with diameters around 100 nm after hydration (Figure 1A).

**FIGURE 1:**
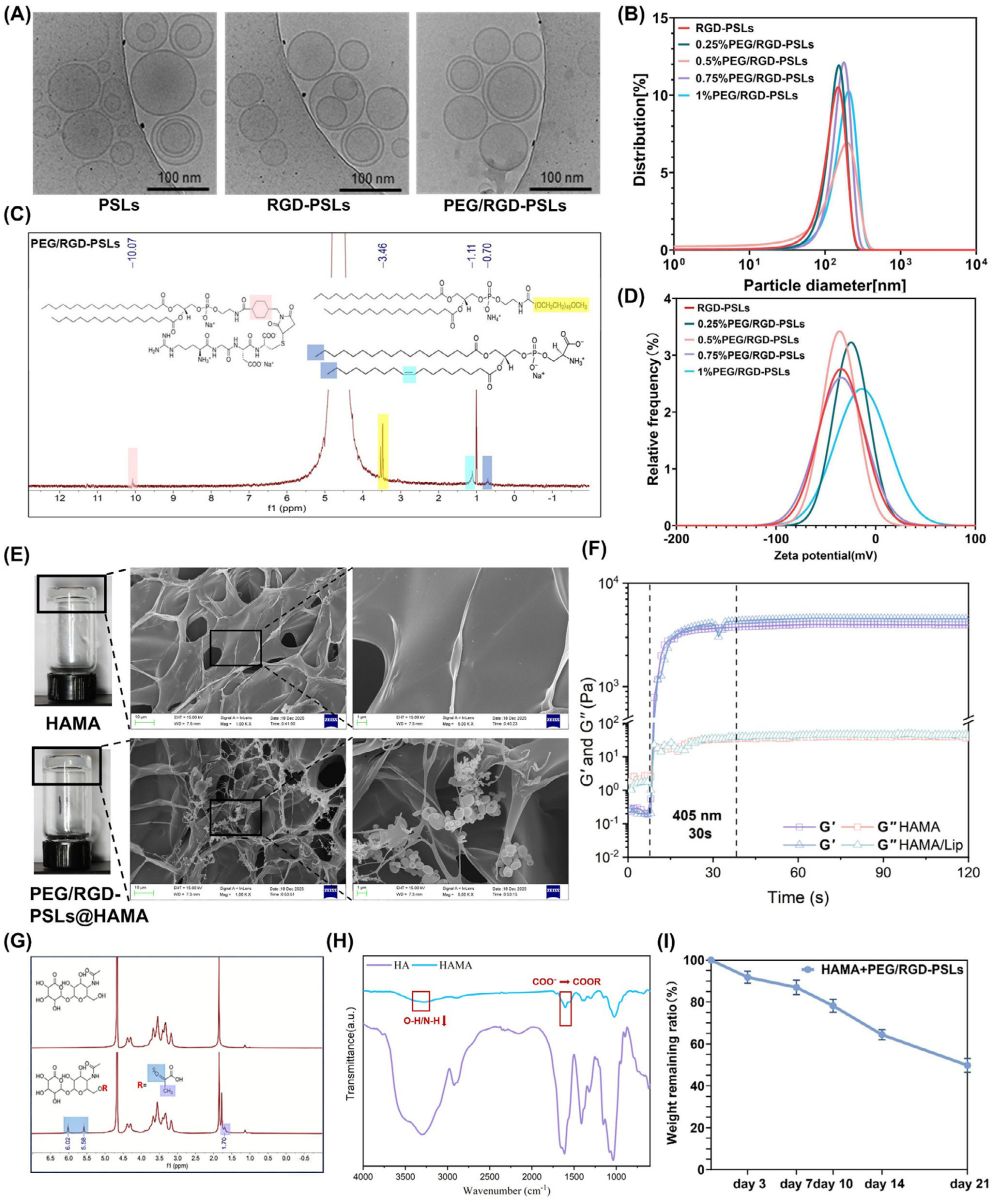
Fabrication and characterization of PEG/RGD‐PSLs and the bioactive HAMA hydrogel. (A) Representative transmission electron microscopy (TEM) image of PEG/RGD‐PSLs (scale bar: 100 nm). (B) Dynamic light scattering (DLS) measurements showing the hydrodynamic diameter of liposomes with increasing PEGylation ratios. (C) ^1^H NMR spectrum confirming successful PEG conjugation on liposomes. (D) Zeta potential of liposome formulations as a function of PEG molar ratio. (E) Scanning electron microscopy (SEM) image of the lyophilized PEG/RGD‐PSLs@HAMA hydrogel, revealing a porous microstructure with integrated liposomes (scale bar: 10 and 1 µm). (F) Real‐time rheological analysis during photocrosslinking, showing the storage modulus (*G*′) and loss modulus (*G*″) for pure HAMA and the PEG/RGD‐PSLs@HAMA composite hydrogel. (G) ^1^H NMR spectrum of hyaluronic acid methacryloyl (HAMA), confirming successful methacrylation. (H) Fourier‐transform infrared (FTIR) spectra of native HA and HAMA. (I) In vitro degradation profile of the PEG/RGD‐PSLs@HAMA hydrogel in PBS over 21 days (mean ± SD, *n* = 3).

The hydrogel matrix was fabricated from methacrylated hyaluronic acid (HAMA). Successful methacrylation was verified by ^1^H‐NMR (Figure 1G), where new proton peaks corresponding to the methacrylate group appeared at 1.70, 5.58, and 6.02 ppm. Fourier‐transform infrared (FTIR) (Figure 1H) spectroscopy provided further evidence: a weakened absorption at 3300 cm^−^^1^ indicated the consumption of hydroxyl groups during esterification, while a reduced signal at 1600 cm^−^^1^ suggested the conversion of carboxylate ions (COO^−^) to esters (COOR), collectively confirming the successful introduction of methacrylate functionalities. The photocrosslinkable precursor solution, containing HAMA and the optimally selected 0.5%PEG/RGD‐PSLs, formed a transparent, cohesive hydrogel within 30 s of UV exposure (405 nm). Real‐time rheological analysis during photocrosslinking revealed a distinct three‐phase sol–gel transition. In the initial phase (0–8 s, no UV), the loss modulus (*G″*) exceeded the storage modulus (*G*′), characteristic of a viscous solution. Upon UV initiation (8 s), both moduli increased sharply, with *G*′ surpassing *G*″ at the gelation point, indicating rapid and efficient free‐radical polymerization and network formation. In the final plateau phase (38–120 s, post‐UV), the moduli stabilized, confirming the formation of a stable viscoelastic solid. Notably, the final equilibrium storage modulus (*G*′) of the HAMA/liposome composite was higher than that of the pure HAMA hydrogel, demonstrating that the incorporated liposomes not only did not hinder photocrosslinking but actively enhanced the mechanical rigidity of the resulting network (Figure 1F). Scanning electron microscopy (SEM) of the lyophilized hydrogel revealed a highly porous and interconnected microstructure, with liposomes distributed and integrated within the polymer matrix (Figure 1E). In vitro degradation studies demonstrated sustained mass loss over 21 days, with approximately 50.6% of the initial mass remaining (Figure 1I).

### 2.2 PEG/RGD‐PSLs Enhance Targeted Uptake and Promote Macrophage Recruitment

To evaluate the targeting efficiency and immunomodulatory potential of the liposome formulations, we first assessed their cellular uptake by macrophages under diabetic‐mimicking (high glucose + LPS) conditions. Flow cytometry and confocal microscopy analyses revealed distinct, time‐dependent uptake kinetics for the different formulations.

Within the first hour, RGD‐PSLs exhibited the most rapid uptake (54.85% ± 1.09% positive cells), significantly outperforming non‐PS control liposomes (PCLs, 27.04% ± 2.13%), underscoring the contribution of PS within the RGD‐functionalized formulation. PEGylation altered this profile in a concentration‐dependent manner, reducing initial (3‐h) uptake—consistent with its stealth properties—while enabling a more gradual and sustained increase in signal intensity over 12 h. By 12–24 h, uptake reached saturation across all groups (Figure 2A,B). Confocal microscopy visually corroborated these differential association patterns (Figure 2C,D).

FIGURE 2PEG/RGD‐PSLs enhance targeted uptake and promote macrophage recruitment in vitro. (A and B) Flow cytometry analysis of cellular uptake kinetics of fluorescently labeled liposomes by RAW 264.7 macrophages under diabetic‐mimicking (HG + LPS) conditions over 24 h. (A) Percentage of fluorescence‐positive cells. (B) Mean fluorescence intensity. (C and D) Representative confocal microscopy images of macrophages after 3 h (C) and 12 h (D) of incubation with TRITC‐labeled liposomes (red). Cell membranes are stained with DiOC_18_ (3) (green). Scale bars: 50 µm. (E) Schematic of the Transwell migration assay. (F) Representative microscopy images of macrophages after 12 h of incubation with liposome‐conditioned medium. Scale bars: 200 µm. (G) Quantitative analysis of macrophage migration. Both M1‐ and M2‐polarized macrophages showed enhanced migration toward PEG/RGD‐PSLs compared to PCLs controls. Data are mean ± SD (*n* = 3); *p < 0.05, **p < 0.01, ***p < 0.001.

**Figure:**
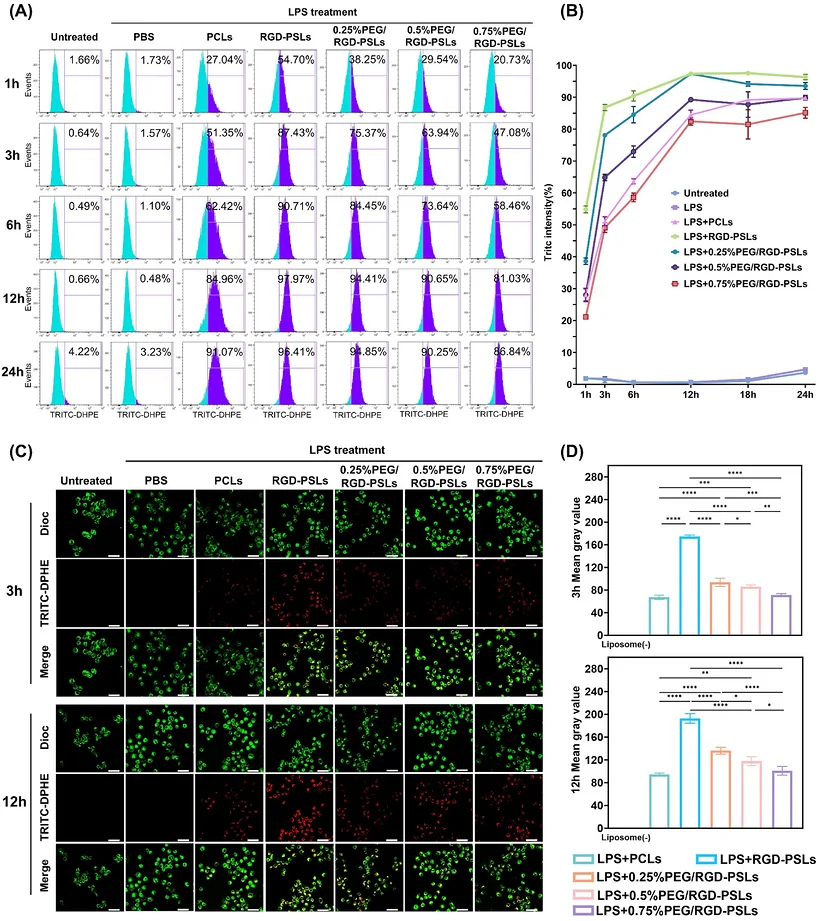

**Figure:**
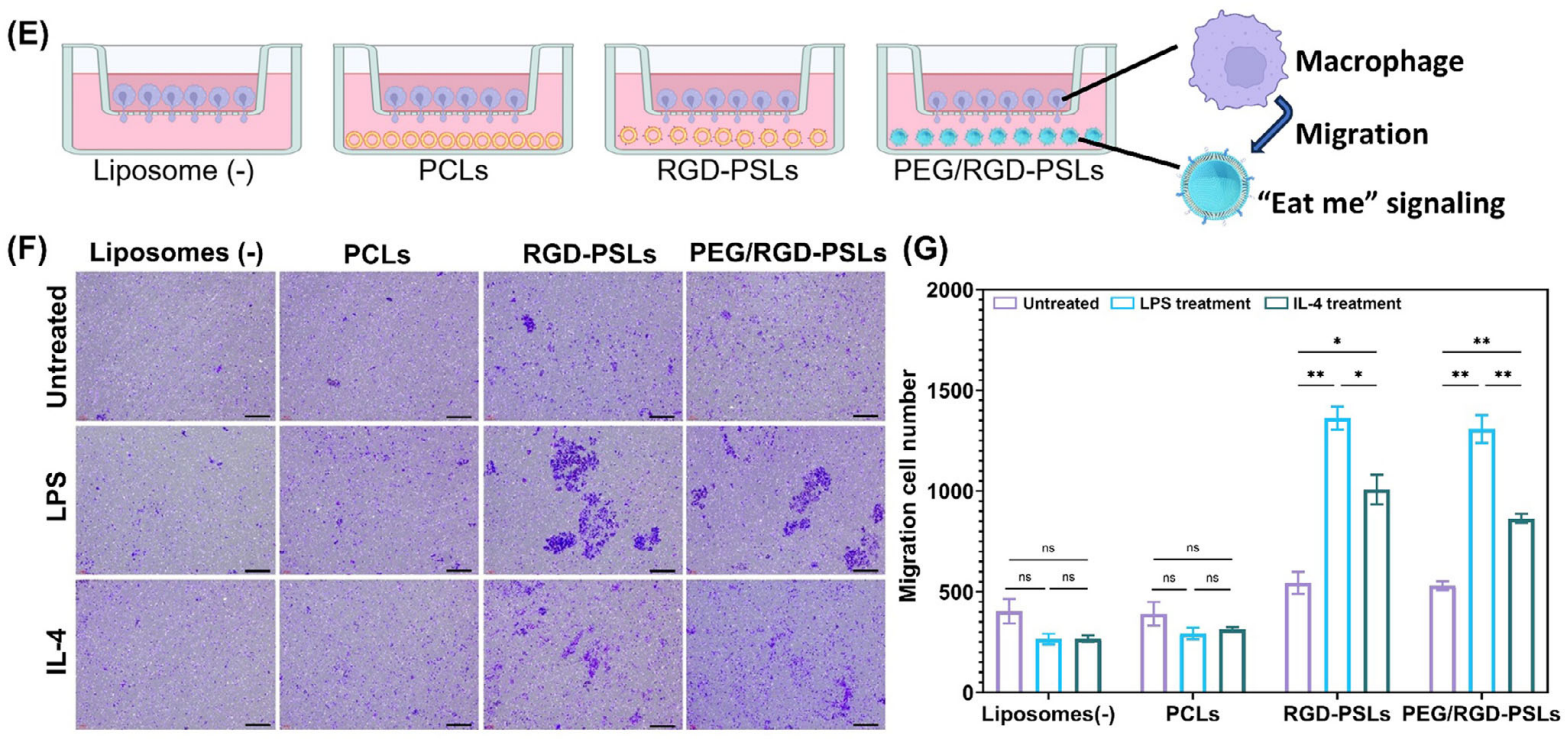

The functional consequence of this enhanced and sustained cellular engagement was assessed by evaluating macrophage migration—a critical early step in wound healing. Conditioned medium containing either RGD‐PSLs or the optimal PEG/RGD‐PSLs formulation potently stimulated the migration of both M1‐polarized macrophages (RGD‐PSLs: 1362 ± 57.26; PEG/RGD‐PSLs: 1307 ± 69.01) and M2‐polarized macrophages (RGD‐PSLs: 1007 ± 73.55; PEG/RGD‐PSLs: 863 ± 23.18) in a Transwell assay, a finding significantly superior to that of the PCLs control (M1: 293 ± 28.73; M2: 314 ± 11.14) (Figure 2F,G). This demonstrates that the engineered liposomes not only achieve efficient and tunable cellular uptake but also actively promote a key reparative response: the targeted recruitment of macrophages to the wound site. These findings establish that PEG/RGD‐PSLs can strategically overcome the impaired chemotaxis characteristic of diabetic macrophages, providing a foundation for their subsequent immunomodulatory functions.

### 2.3 PEG/RGD‐PSLs Reprogram Macrophage Polarization and Confer Cytoprotection

We next investigated the capacity of liposome formulations to modulate macrophage phenotype under sustained diabetic stress (HG + LPS). A time‐course analysis across 3, 6, 12, and 24 h revealed that PEGylation critically optimized the anti‐inflammatory profile of RGD‐PSLs, shifting the efficacy from an immediate to a durable response.

#### 2.3.1 PEGylation Enables Sustained Anti‐Inflammatory Efficacy

RT‐qPCR demonstrated that both RGD‐PSLs and PEG/RGD‐PSLs effectively suppressed LPS‐induced M1 polarization, as evidenced by significant downregulation of key inflammatory genes (iNOS, TNF‐α, IL‐6, IL‐1β) compared to the LPS control (Figure 3A–D). However, the efficacy was strikingly time‐dependent. In the early phase (3–6 h), RGD‐PSLs exerted a more potent immediate effect. For instance, at 3 h, RGD‐PSLs reduced IL‐1β expression from 208.04 ± 14.24‐fold (LPS) to 35.70 ± 7.49‐fold, whereas 0.5%PEG/RGD‐PSLs reduced it to 84.4 ± 13.89‐fold. Similar trends were observed for iNOS (3 h) and TNF‐α (6 h).

FIGURE 3PEG/RGD‐PSLs reprogram macrophage polarization under diabetic‐mimicking stress. (A–D) Time‐course RT‐qPCR analysis of M1 marker gene expression (iNOS, TNF‐α, IL‐6, IL‐1β) in macrophages treated with LPS and different liposome formulations. (E–H) RT‐qPCR analysis of M2 marker gene expression (Arg‐1, CD206, YM‐1, FIZZ‐1) after treatment of pre‐polarized M1 macrophages with liposomes. (I, M, J, O) Flow cytometry quantification of iNOS^+^ (I and M) and Arg‐1^+^ (J and O) macrophage populations. (K and L) Representative immunofluorescence images for iNOS (K, red) and Arg‐1 (L, red) expression. Nuclei are stained with DAPI (blue). Scale bars: 100 µm. (N and P) Corresponding quantitative analysis of fluorescence intensity from panels K and L. Data are mean ± SD (*n* = 3); *p < 0.05, **p < 0.01, ***p < 0.001.

**Figure:**
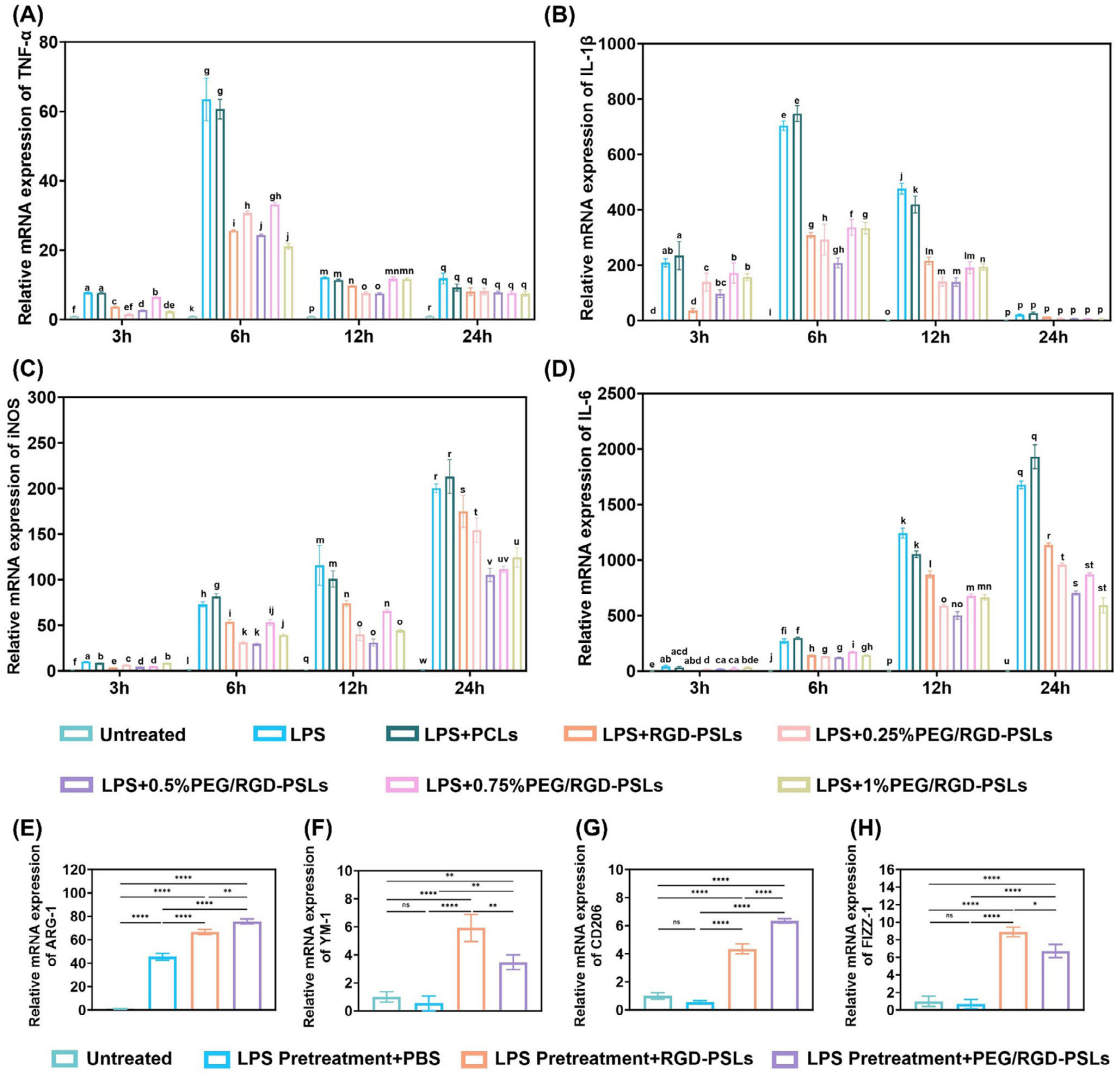

**Figure:**
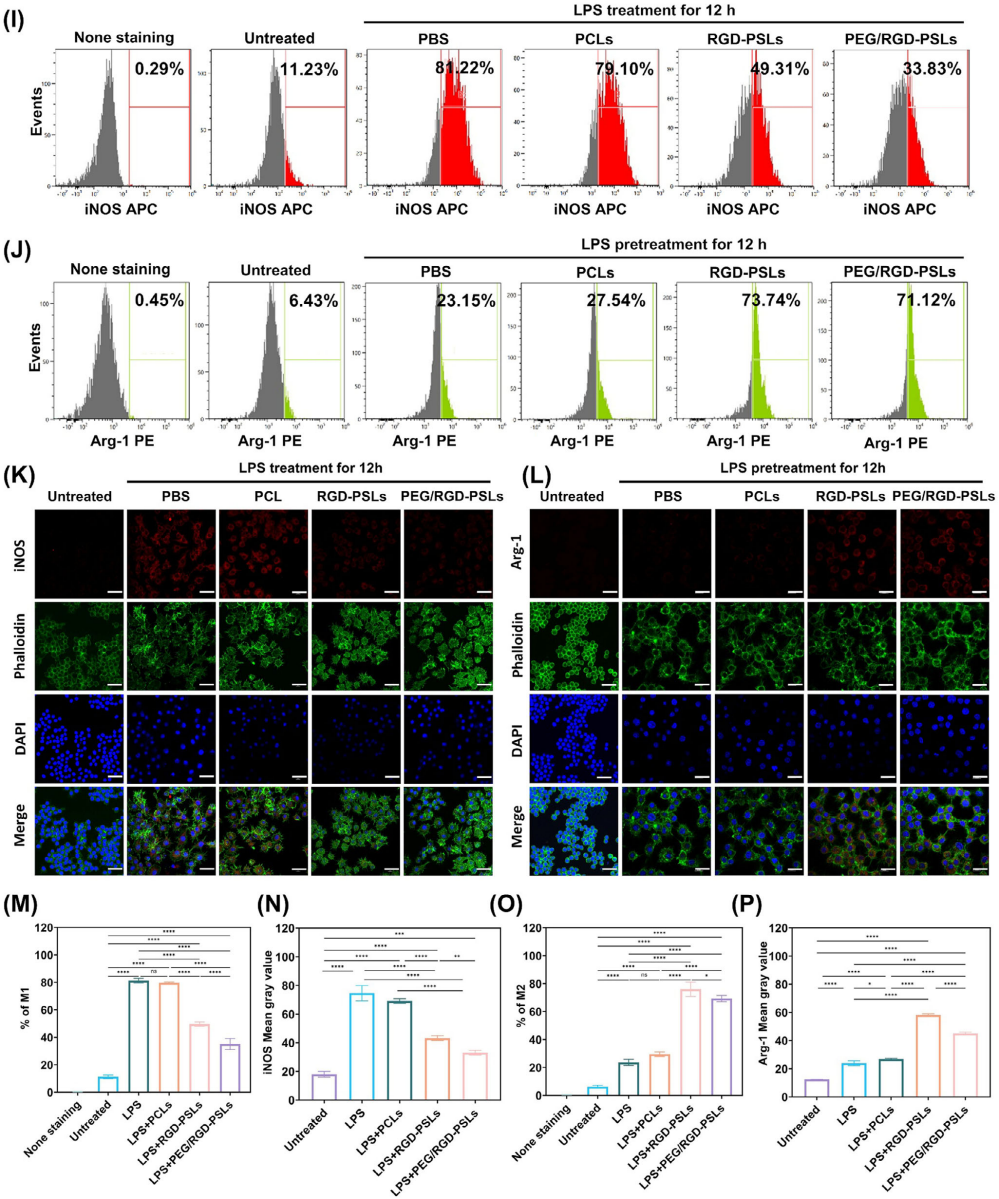

Conversely, as exposure extended, the PEGylated formulations (particularly 0.25% and 0.5% PEG) demonstrated superior and sustained anti‐inflammatory activity. From 6 to 24 h, the expression of IL‐1β, iNOS, and IL‐6 was consistently lower in these groups compared to the RGD‐PSLs group. This shift aligns perfectly with the uptake kinetics (Section 2.2), suggesting that initial PEG‐mediated stealth delays recognition but facilitates prolonged engagement, leading to more durable transcriptional repression. This optimized function was confirmed at the protein level: flow cytometry showed that the percentage of iNOS^+^ cells decreased from 81.22% ± 1.71% (LPS) to 49.53% ± 1.60% (RGD‐PSLs) and further to 35.11% ± 3.98% (PEG/RGD‐PSLs), a significant enhancement (Figure 3I,M). Immunofluorescence staining corroborated this trend, visually affirming that PEGylation augments the anti‐inflammatory functionality of RGD‐PSLs, likely by prolonging their bioactivity (Figure 3K,N).

#### 2.3.2 Repolarization From M1 to a Pro‐Reparative M2 Phenotype

Furthermore, when applied to pre‐polarized M1 macrophages, the optimal 0.5%PEG/RGD‐PSL formulation effectively drove repolarization toward a reparative M2 phenotype. This was evidenced by significant upregulation of M2 markers (Arg‐1, CD206, YM‐1, FIZZ‐1) at the mRNA level (Figure 3E–H) and an increased population of Arg‐1^+^ cells (Figure 3J,L,O,P).

#### 2.3.3 Cytoprotection Under Diabetic Stress Conditions

Notably, beyond phenotypic reprogramming, PEG/RGD‐PSLs conferred profound cytoprotection under HG + LPS stress. SEM analysis revealed a significant preservation of cellular morphology (Figure 4A). LPS stimulation typically induced a dramatic transformation of macrophages from a rounded to a highly spread, flattened morphology with multiple protrusions, accompanied by excessive membrane blebbing and the formation of abnormal extracellular vesicle‐like structures. Treatment with both RGD‐PSLs and PEG/RGD‐PSLs markedly inhibited these pathological morphological changes, maintaining greater cellular integrity and reducing vesiculation (Figure 4A).

FIGURE 4PEG/RGD‐PSLs confer cytoprotection under diabetic stress. (A) Scanning electron microscopy (SEM) images showing macrophage surface morphology under different treatments. PEG/RGD‐PSLs treatment inhibited LPS‐induced membrane blebbing and pathological vesiculation. Scale bars: 10 µm (2000×), 10 µm (20000×). (B) Transmission electron microscopy (TEM) images revealing macrophage ultrastructure. LPS caused severe mitochondrial (Mi) swelling, cristae disruption, outer membrane rupture (blue arrows), and RER dilation. PCLs treatment offered limited protection. In contrast, RGD‑PSLs and PEG/RGD‑PSLs groups preserved normal cellular architecture, including intact nuclei (N), organized mitochondrial cristae (red arrows), Golgi bodies (GB), and RER. Notably, autophagic vacuoles containing liposome‑like structures (green arrows, bright yellow arrows) were abundant in PEG/RGD‑PSLs‑treated cells, indicating successful internalization. Scale bars: 2 µm (6000×), 1 µm (20000×). (C) Flow cytometric measurement of intracellular total ROS levels in macrophages using DCFH‐DA. (D) Proportion of cells with high MitoTracker Green staining. (E) Mitochondrial ROS (mtROS) levels were measured by MitoSOX Red staining. (F and G) Wound closure percentage in the fibroblast scratch assay when co‐cultured with differentially conditioned macrophages. (H) Intracellular ROS levels in NIH/3T3 fibroblasts after co‐culture with conditioned macrophages. Data are mean ± SD (*n* = 3); *p < 0.05, **p < 0.01, ***p < 0.001.

**Figure:**
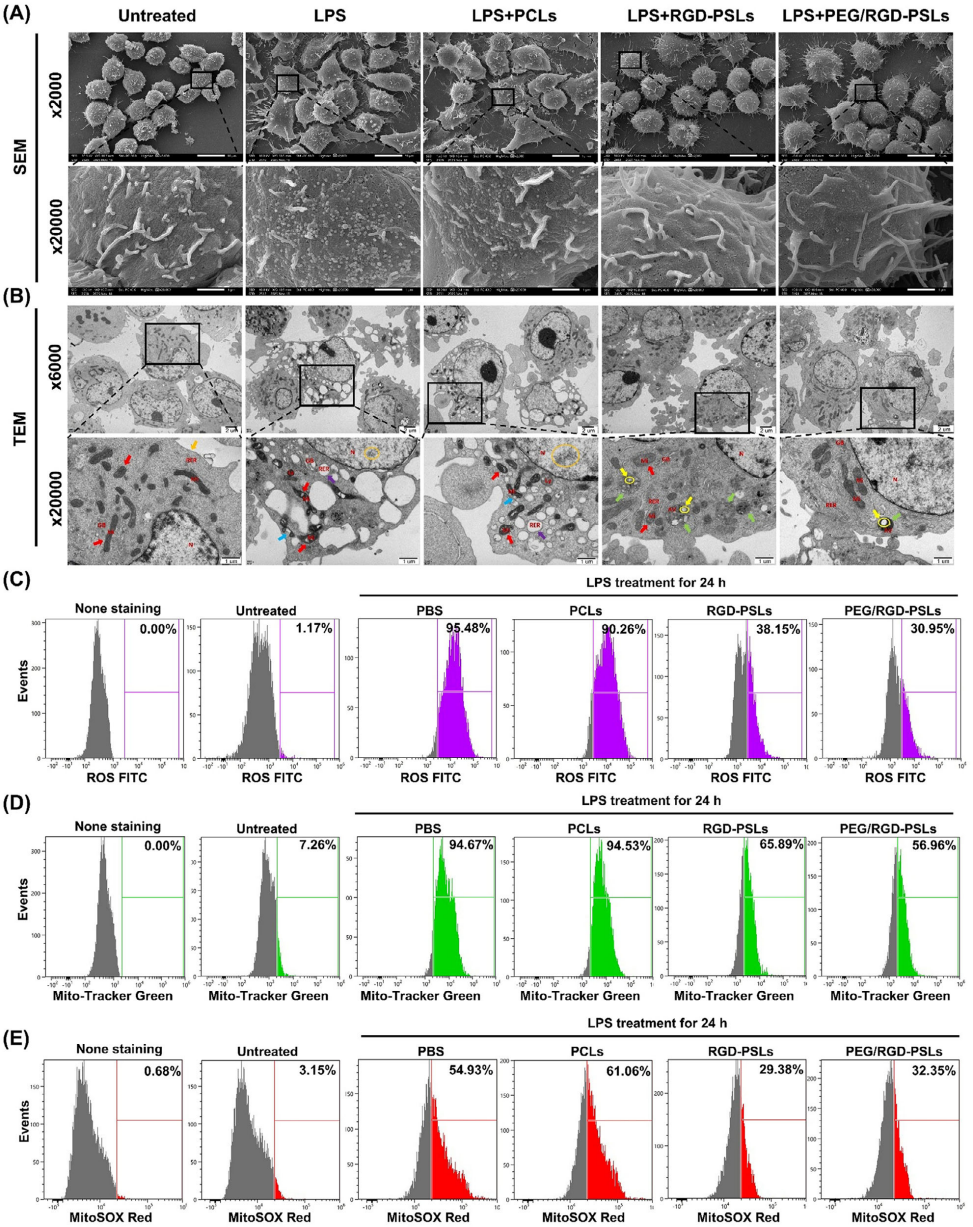

**Figure:**
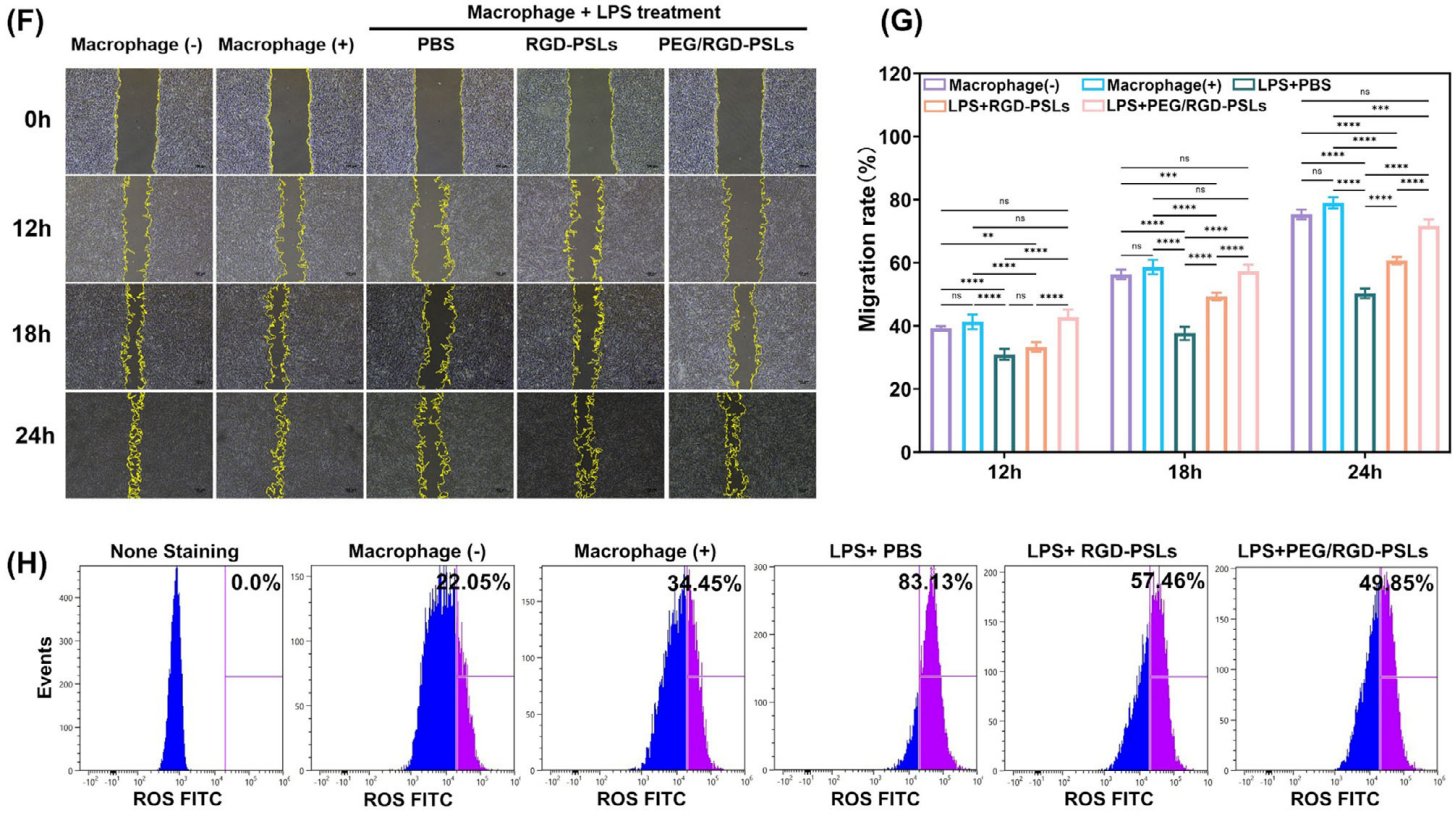

This protection extended to the intracellular ultrastructure, as detailed by TEM (Figure 4B). Macrophages under normal conditions exhibited intact organelles. HG + LPS stress, however, induced severe pathology, including nuclear chromatin condensation, mitochondrial swelling with cristae disruption, and marked dilation of the rough endoplasmic reticulum (RER). Treatment with PCLs failed to rescue these defects. In striking contrast, cells treated with the functional liposomes (RGD‐PSLs and PEG/RGD‐PSLs) showed well‐preserved cellular architecture. Most importantly, their cytoplasm contained numerous autophagic vacuoles and secondary lysosomes enclosing vesicular structures morphologically consistent with internalized liposomes. This provides morphological evidence consistent with intracellular uptake. The efficient cellular uptake of the therapeutic liposomes was thus intrinsically linked to the observed mitigation of ultrastructural damage.

#### 2.3.4 PEG/RGD‐PSLs Treatment Ameliorates LPS‐Induced Oxidative Stress and Mitochondrial Dysfunction in Macrophages

We next investigated whether the observed anti‐inflammatory effects of PEG/RGD‐PSLs were associated with the modulation of intracellular oxidative stress and mitochondrial homeostasis. As quantified by flow cytometry using DCFH‐DA staining, LPS stimulation drastically increased the level of intracellular total ROS from 2.01% ± 1.12% in the control group to 83.89% ± 12.82% (Figures 4C and S2A). Treatment with PEG/RGD‐PSLs significantly attenuated this oxidative burst, reducing the ROS‐positive cell population to 29.56% ± 3.41% (Figures 4C and S2A). A similar trend was observed specifically for mitochondrial ROS (mtROS), detected using MitoSOX Red. LPS stimulation elevated the mtROS‐high population to 54.28% ± 0.59% compared to 3.05% ± 0.66% in control cells, and this increase was mitigated to 34.97% ± 2.50% upon PEG/RGD‐PSLs treatment (Figures 4E and S2C).

Consistent with the reduction in mtROS, PEG/RGD‐PSLs treatment preserved mitochondrial structural integrity. TEM analysis revealed that LPS stimulation induced marked mitochondrial swelling and cristae disruption. In contrast, mitochondria in PEG/RGD‐PSLs‐treated cells maintained a normal morphology, comparable to that of the control group (Figure 4B). This morphological rescue correlated with functional metrics. Staining with Mito–Tracker Green showed that the proportion of cells with high fluorescence intensity increased to 94.67% ± 0.39% after LPS exposure (compared with 2.01% ± 1.12% in controls). PEG/RGD‐PSLs treatment effectively reduced this proportion to 57.36% ± 2.36% (Figures 4D and S2B).

Corroborating these phenotypic observations at the transcriptional level, RNA‐sequencing analysis revealed that PEG/RGD‐PSLs treatment significantly modulated pathways related to oxidative stress. Most notably, there was a marked enrichment in the gene set associated with “cellular response to oxidative stress” when comparing the PEG/RGD‐PSLs treatment group to the LPS‐stimulated group (Figure S2E,F). This regulation of antioxidant defense genes provides a molecular mechanistic basis for the substantial attenuation of intracellular and mitochondrial ROS levels documented by our flow cytometry data.

#### 2.3.5 Enhanced Pro‐Healing Paracrine Function

This cellular resilience translated into functional enhancement in a macrophage‐fibroblast co‐culture system, where macrophages conditioned with PEG/RGD‐PSLs significantly accelerated the migration and scratch closure of NIH/3T3 fibroblasts, outperforming those conditioned with M1 macrophages (71.67% ± 2.08% vs. 47.00% ± 4.36%) (Figure 4F,G). Critically, this enhanced reparative output was accompanied by a substantial alleviation of oxidative stress in the fibroblasts. Flow cytometric analysis revealed that intracellular ROS levels in fibroblasts exposed to the conditioned medium from PEG/RGD‐PSL‐treated macrophages were reduced to 47.05% ± 1.58%, compared to 80.86% ± 7.06% in the LPS (M1) group and 34.16% ± 0.44% in the untreated group (Figures 4H and S2D). These results confirm that our intervention not only reprograms and protects macrophages at a cellular level but also functionally augments their capacity to support tissue regeneration by promoting fibroblast migration and mitigating the detrimental oxidative microenvironment.

### 2.4 Transcriptomic Profiling Elucidates the Mechanism of Macrophage Reprogramming via PS/CD300a‐Mediated Suppression of NF‐κB Signaling

To elucidate the mechanisms underlying the phenotypic reprogramming by PEG/RGD‐PSLs, we performed RNA sequencing on macrophages under diabetic‐mimicking stress (HG + LPS). Principal component analysis (PCA) revealed a clear transcriptomic divergence induced by HG + LPS, which was substantially reversed by PEG/RGD‐PSL treatment, shifting the profile back toward the normal state (Figure S3A). Differential expression analysis identified 2905 genes significantly dysregulated by HG + LPS stimulation (vs. NC; |log2FC| ≥ 1, Q ≤ 0.05) (Figure S3B). Treatment with PEG/RGD‐PSLs specifically rescued the expression of 333 of these stress‐responsive genes (Figure S3B). Specifically, HG + LPS induced the upregulation of 1600 genes and the downregulation of 1305 genes (Figure S3C; volcano plot a in Figure S3D). KEGG pathway enrichment and gene set enrichment analysis (GSEA) of these DEGs confirmed the hyperactivation of key inflammatory pathways, including NF‐κB and TNF signaling (Figure S3E,F).

Critically, analyzing the genes reversed by PEG/RGD‐PSLs treatment (LPS + PEG/RGD‐PSLs vs. LPS) revealed a distinct anti‐inflammatory signature (164 up, 169 down; Figure S3I, volcano plot in Figure 5A). KEGG and GSEA demonstrated that PEG/RGD‐PSLs significantly suppressed inflammatory pathways activated by HG + LPS, most notably the NF‐κB signaling pathway (Figure 5B,E). This transcriptional repression was validated at the protein level, as Western blot analysis demonstrated that PEG/RGD‐PSLs significantly downregulated the expression and phosphorylation of key NF‐κB pathway components (MyD88, phospho‐MyD88, total p65, and phospho‐p65) at 3 and 12 h post‐treatment (Figure 5F). A comprehensive KEGG network and GSEA analysis further highlighted the broad downregulation of inflammation‐associated pathways, including cytokine‐cytokine receptor interaction, the TNF signaling pathway, the JAK‐STAT signaling pathway, and the PI3K‐Akt signaling pathway (Figure 5D,E). Concurrently, PEG/RGD‐PSLs treatment was associated with altered expression of multiple genes involved in metabolic pathways crucial for cellular energy homeostasis, including oxidative phosphorylation, the citrate cycle (TCA cycle), and carbon metabolism (Figure S3G,H), suggesting potential metabolic reprogramming that warrants further investigation.

FIGURE 5Transcriptomic analysis reveals PEG/RGD‐PSLs suppress inflammatory pathways. (A) Volcano plot of differentially expressed genes (DEGs) in macrophages treated with PEG/RGD‐PSLs versus LPS stimulation under diabetic‐mimicking conditions (HG + LPS). (B) KEGG pathway enrichment analysis of DEGs reversed by PEG/RGD‐PSLs treatment. (C) Heatmap of selected key inflammatory genes downregulated by PEG/RGD‐PSLs treatment. (D) Comprehensive network visualization of KEGG pathways suppressed by PEG/RGD‐PSLs. (E) Gene Set Enrichment Analysis (GSEA) plots demonstrating the downregulation of hallmark inflammatory pathways, including NF‐κB signaling. (F) Western blot analysis confirming PEG/RGD‐PSLs‐mediated downregulation of MyD88, phospho‐MyD88, total p65, and phospho‐p65 at 3 and 12 h. Data are mean ± SD (*n* = 3); *p < 0.05, **p < 0.01, ***p < 0.001.

**Figure:**
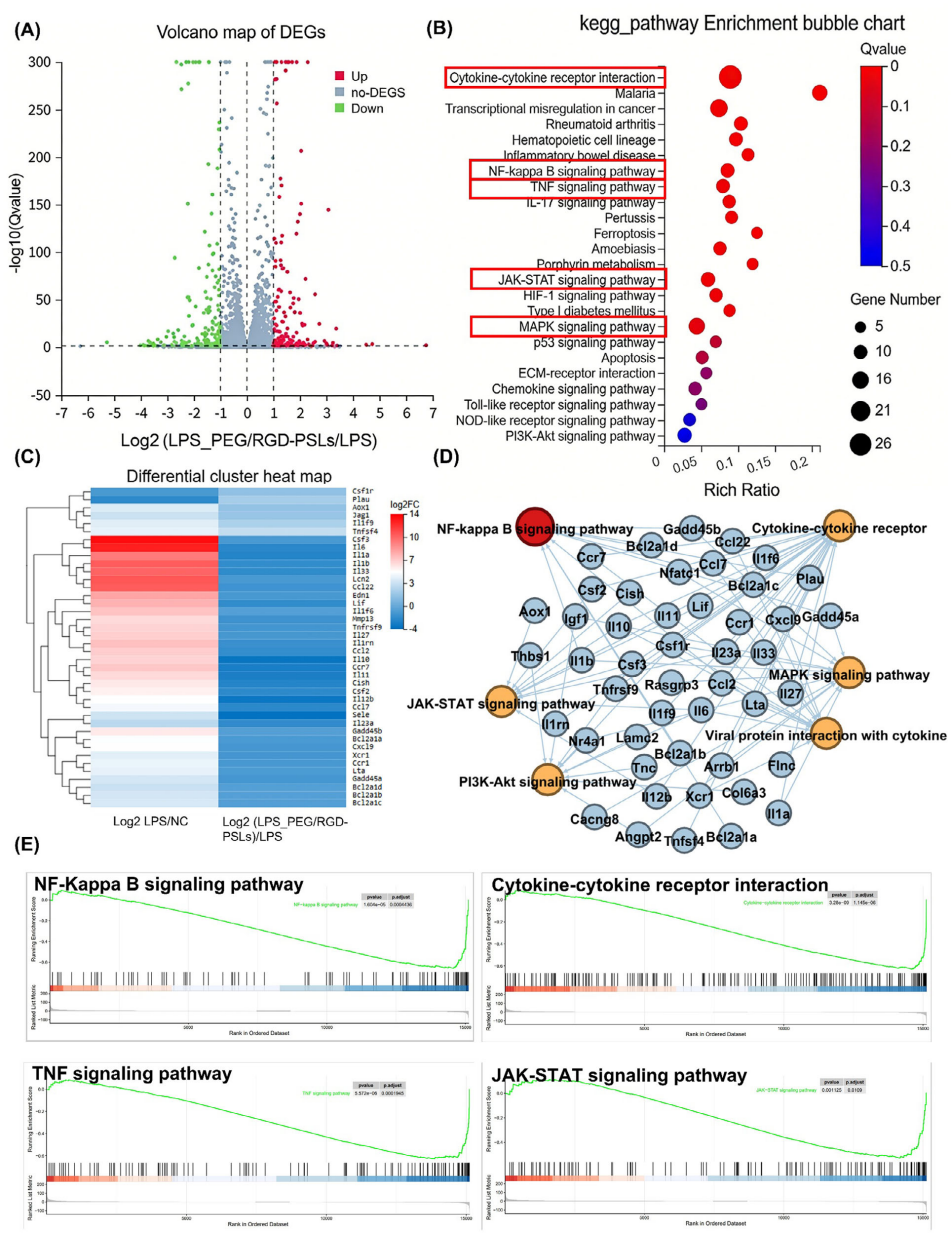

**Figure:**
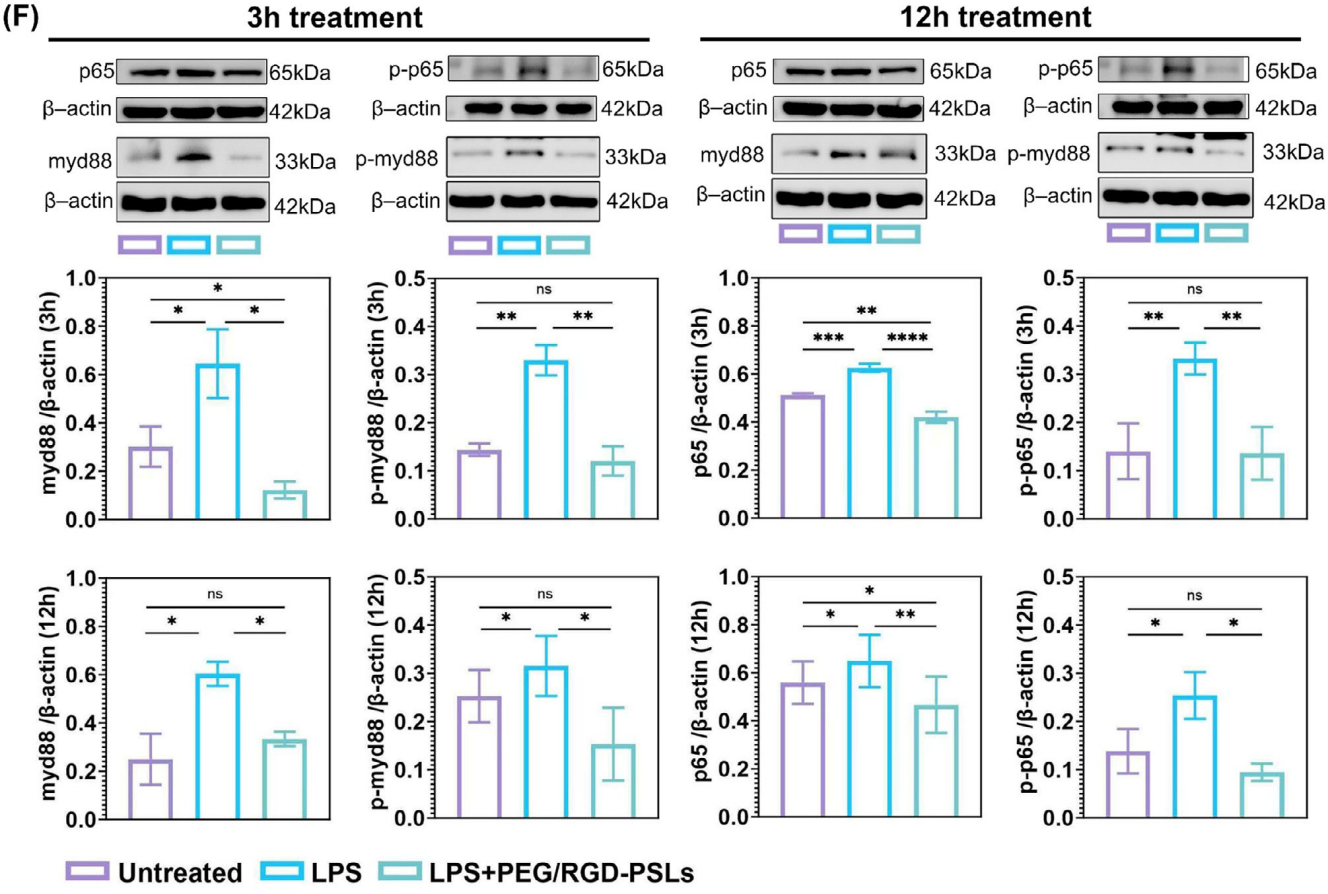

Given that the liposomes mimic apoptotic cells via surface PS, we investigated the engagement of PS receptors. Molecular function (GO) enrichment analysis of PEG/RGD‐PSLs‐responsive genes revealed significant enrichment for “phosphatidylserine binding” (Figure 6A). Among the regulated PS‐binding receptors, the gene encoding the inhibitory receptor CD300a was significantly upregulated, while others such as Trem2 and Mfge8 showed similar trends, and genes like Marcks, Havcr1, Thbs1, and Syt7 were downregulated (Figure 6B,C,E). RT‑qPCR validation (Figure S4) revealed that only CD300a exhibited statistically significant upregulation upon PEG/RGD‑PSL treatment. Protein–protein interaction (PPI) and bioinformatic network analyses positioned CD300a as a central node negatively correlated with pro‐inflammatory pathways, including NF‐κB signaling (Figure 6D,G). Western blot analysis confirmed the upregulation of CD300a protein expression upon PEG/RGD‐PSL treatment (Figure 6F). This aligns with the established biology wherein PS binding to CD300a transduces inhibitory signals that dampen inflammatory responses [38, 39].

FIGURE 6PEG/RGD‐PSLs engage the PS receptor CD300a to mediate anti‐inflammatory effects. (A) Gene Ontology (GO) molecular function enrichment analysis, highlighting “phosphatidylserine binding” as a top‐ranked term. (B) Volcano plot of DEGs, with labeled points representing key phosphatidylserine‐binding receptor genes (e.g., Cd300a, Trem2). (C) Heatmap depicting the expression profile of phosphatidylserine‐binding receptor genes regulated by PEG/RGD‐PSLs. (D) Protein–protein interaction (PPI) network with CD300a as a central node, illustrating its connectivity to inflammatory gene modules. (E) Normalized expression (FPKM) of CD300a across treatment groups. (F) Western blot analysis confirming PEG/RGD‐PSLs‐mediated upregulation of CD300a protein and downregulation of MyD88, phospho‐MyD88, total p65, and phospho‐p65 at 3 and 12 h. (G) Bioinformatic network analysis positioning CD300a as a central, negatively correlated node with pro‐inflammatory pathways, including NF‐κB signaling. Data are mean ± SD (*n* = 3); *p < 0.05, **p < 0.01, ***p < 0.001.

**Figure:**
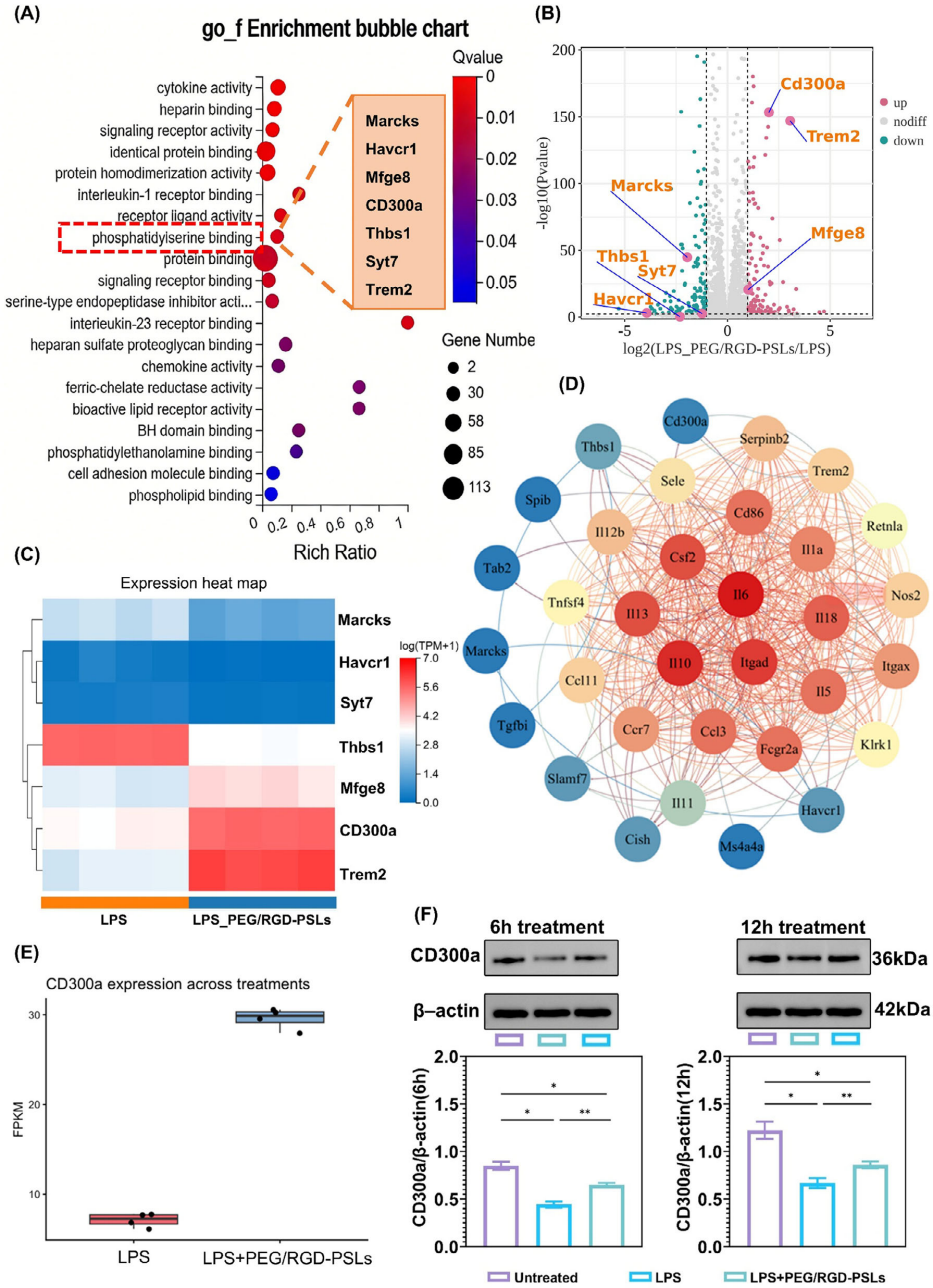

**Figure:**
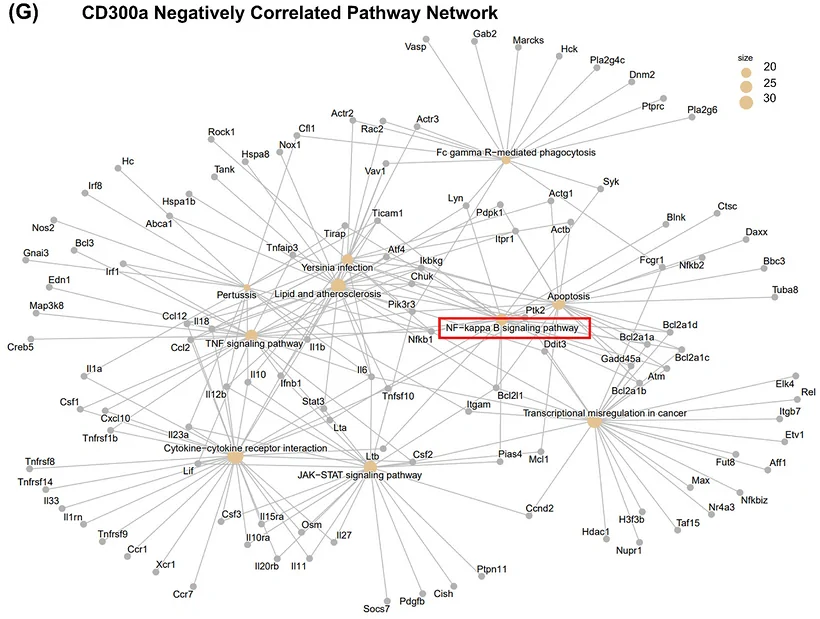

In summary, transcriptomic profiling elucidates that PEG/RGD‐PSLs comprehensively reprogram the diabetic macrophage transcriptome. The core mechanism involves engagement of the inhibitory PS receptor CD300a, leading to suppression of the master inflammatory regulator NF‐κB pathway and a consequent shift from a pro‐inflammatory to a pro‐reparative molecular landscape, providing a systems‐level explanation for the observed in vitro functional benefits.

### 2.5 CD300a Knockdown Abrogates the Anti‑Inflammatory and NF‑κB‑Suppressive Effects of PEG/RGD‑PSLs

To functionally validate that CD300a is the critical receptor mediating the anti‑inflammatory actions of PEG/RGD‑PSLs, we performed CD300a knockdown experiments in RAW 264.7 macrophages under diabetic‑mimicking conditions (high glucose + LPS). Transfection with either of two independent siRNAs targeting CD300a (si‑CD300a‑188 and si‑CD300a‑756) reduced CD300a mRNA levels by over 80% compared with negative control siRNA (si‑NC) (Figure 7A).

**FIGURE 7:**
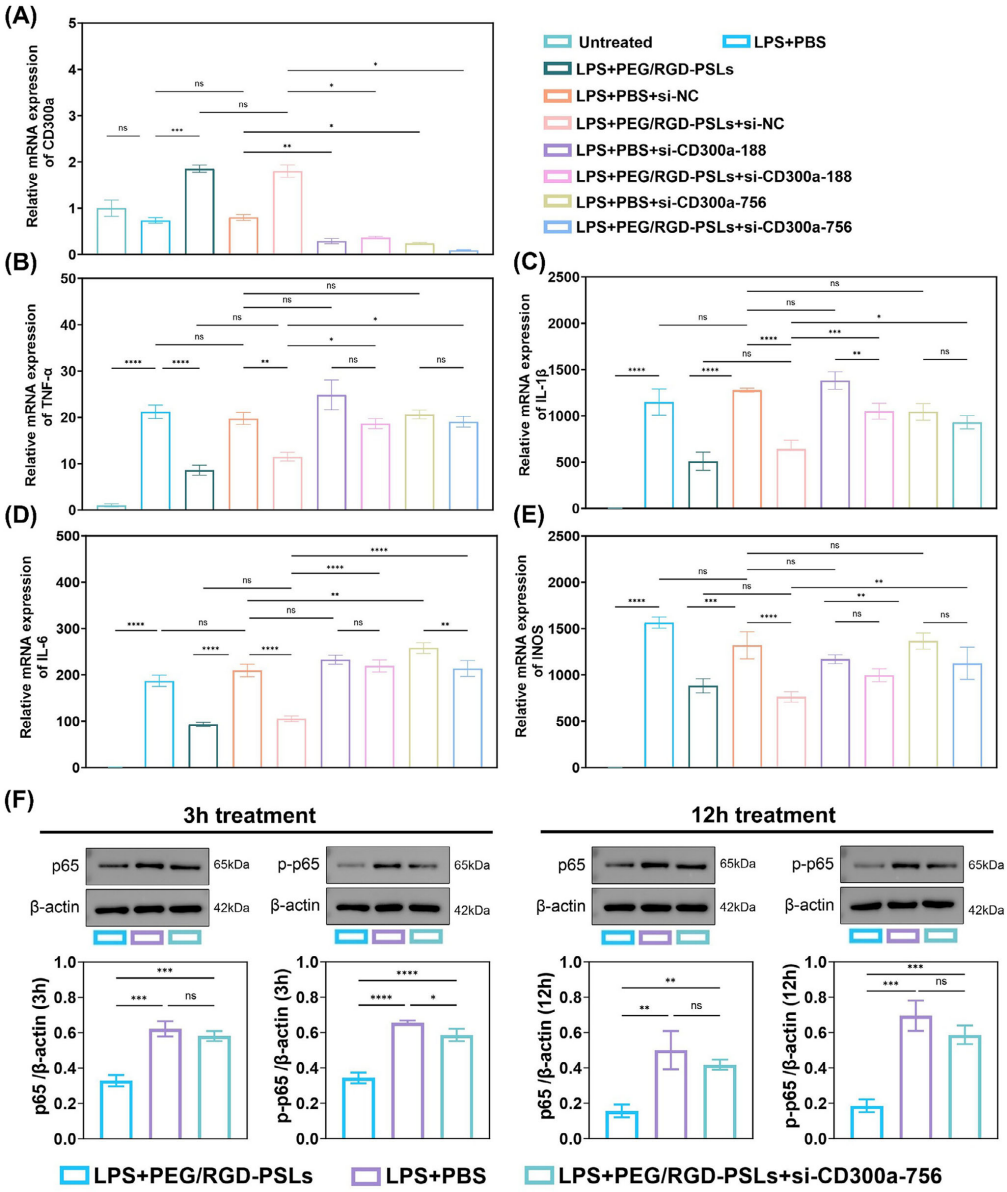
CD300a knockdown abolishes the anti‑inflammatory and NF‑κB‑suppressive effects of PEG/RGD‑PSLs. (A) RT‑qPCR analysis of CD300a mRNA expression 24 h after transfection with si‑NC, si‑CD300a‑188, or si‑CD300a‑756. (B–E) RT‑qPCR analysis of TNF‑α (B), Il‑1β (C), Il‑6 (D), and iNOS (E) mRNA expression after treatment with either si‑CD300a sequence. (F) Representative Western blots of p‑p65 and total p65 after CD300a knockdown with si‑CD300a‑756. Corresponding quantification of p‑p65 and total p65 is also shown. Data are mean ± SD (*n* = 3 independent experiments). *p < 0.05, **p < 0.01, ***p < 0.001. n.s., not significant.

In si‑NC‑transfected cells, PEG/RGD‑PSLs treatment strongly suppressed LPS‑induced upregulation of the pro‑inflammatory cytokines Il‑1β, TNF‑α, Il‑6, and iNOS (Figure 7B–E), consistent with the results in Figure 3. In striking contrast, CD300a knockdown almost completely abolished this anti‑inflammatory effect: the mRNA levels of all four genes remained similarly elevated as in the LPS‑treated and si‑NC groups. Importantly, the si‑NC + PEG/RGD‑PSLs group retained full responsiveness, supporting that the response was not attributable to nonspecific siRNA/transfection effects.

Mechanistically, Western blot analysis showed that PEG/RGD‑PSLs significantly reduced the phosphorylation of NF‑κB p65 (p‑p65) and total p65 expression (Figure 7F). However, CD300a silencing markedly abolished the ability of PEG/RGD‑PSLs to suppress p‑p65 and total p65 levels, indicating that the inhibitory signal downstream of PS engagement cannot be transduced in the absence of CD300a. Collectively, these loss‑of‑function experiments provide direct causal evidence that CD300a is both necessary for PEG/RGD‑PSLs to inhibit the MyD88/NF‑κB pathway and resolve the inflammatory phenotype of diabetic macrophages.

### 2.6 The Bioactive Hydrogel Accelerates Healing in a Diabetic Rat Wound Model

The therapeutic efficacy of the integrated platform was ultimately validated in vivo using a streptozotocin‐induced diabetic rat model of full‐thickness excisional wounds. A single application of the PEG/RGD‐PSLs@HAMA hydrogel dramatically accelerated wound closure, achieving 80.32% ± 4.18% closure by day 10 versus 65.36% ± 5.70% in the sham group, and near‐complete closure (98.73% ± 0.8%) by day 14 (Figure 8A). Histological analysis on days 3 and 7 provided insights into the healing quality. Hydrogel‐treated wounds exhibited accelerated granulation tissue formation, enhanced angiogenesis, and more organized collagen deposition by Masson's trichrome staining compared to both control groups (Figure 8B–E). Time‑course histological analysis revealed that the PEG/RGD‑PSLs@HAMA hydrogel significantly promoted re‑epithelialization in diabetic wounds (Figure S5). At day 3, no epithelial coverage was observed in any group. By day 7, the PEG/RGD‐PSLs‑treated group exhibited a continuous and multilayered epithelial sheet, whereas the sham and blank HAMA groups showed only sporadic and discontinuous epithelial cell clusters. At day 14, all wounds achieved complete epithelial coverage; however, only the PEG/RGD‐PSLs‑treated group displayed mature rete peg‑like invaginations, indicating superior epidermal‑dermal interface integrity. Collectively, these findings demonstrate that the bioactive hydrogel not only accelerates the rate of wound closure but also enhances the quality of epithelial regeneration, promoting the formation of a mature, well‑anchored epidermis.

**FIGURE 8:**
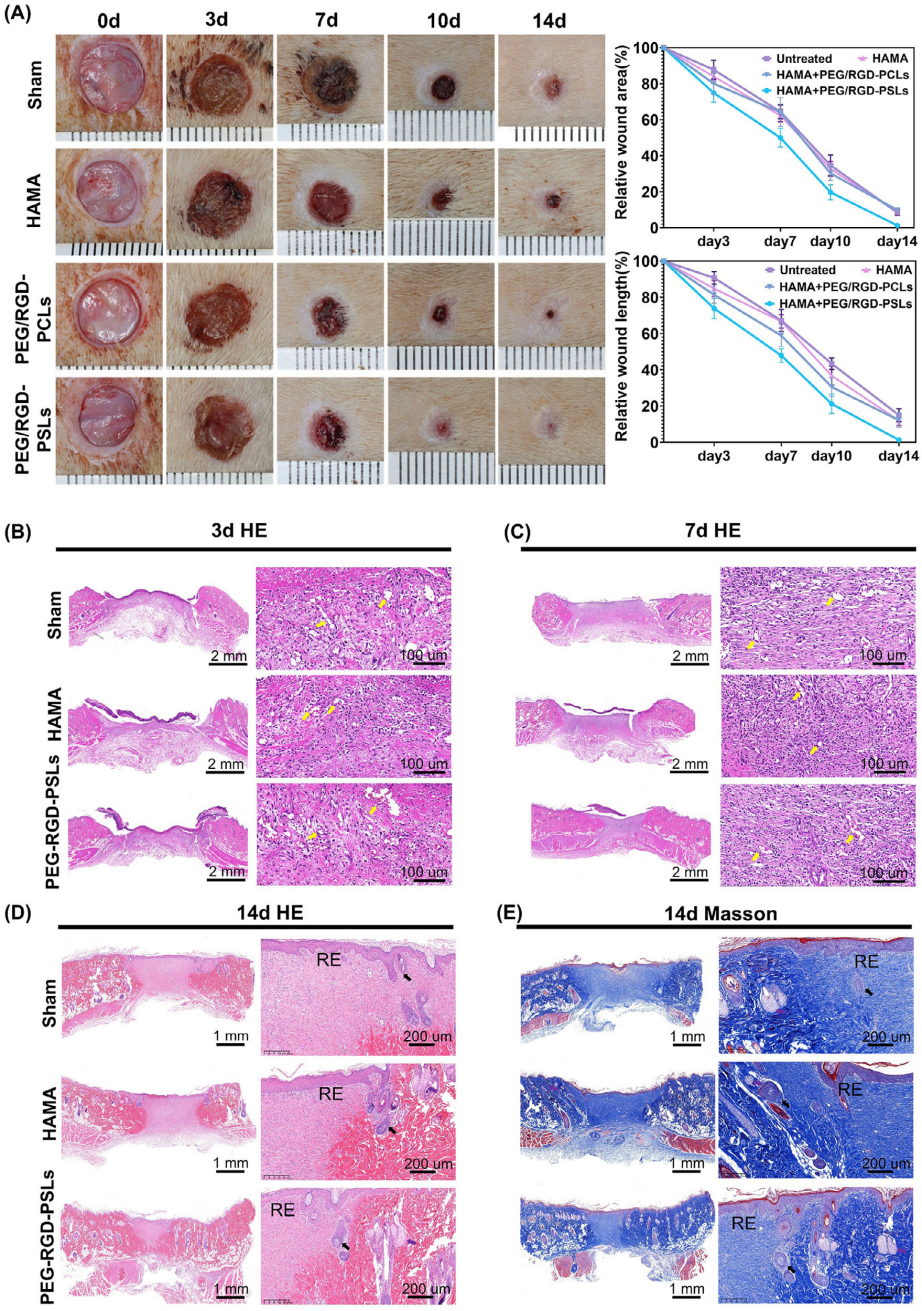
The bioactive hydrogel accelerates diabetic wound healing in vivo. (A) Gross photographs of full‐thickness excisional wounds in diabetic rats across the four treatment groups over 14 days, with corresponding quantification of relative wound area and wound length (mean ± SD, *n* = 6). (B–D) Representative H and E‐stained wound sections from the Sham, HAMA, and PEG/RGD‐PSLs@HAMA groups at day 3 (B), day 7 (C), and day 14 (D), illustrating granulation tissue (GT) formation and re‐epithelialization (RE). Key structures are indicated: blood vessels (yellow arrows), sebaceous glands (purple arrows), and hair follicles (black arrows). (E) Representative Masson's trichrome‐stained sections at day 14, showing collagen deposition (blue). Scale bars: 2 mm and 100 µm in (B and C), and 1 mm and 200 µm in (D and E).

Immunohistochemical (IHC) analysis revealed a pivotal, time‐dependent modulation of the wound immune microenvironment. On day 3, wounds treated with the bioactive hydrogel showed a significant increase in total macrophage (CD68^+^) infiltration (Sham: 121 ± 7.29, HAMA: 120 ± 5.98, PEG/RGD‐PSLs@HAMA: 140 ± 12.3) (Figure 9A), likely attributable to the sustained release of chemotactic PEG/RGD‐PSLs during HAMA degradation. Crucially, further analysis of macrophage subpopulations demonstrated that this enhanced recruitment was coupled with active phenotypic reprogramming. Although total macrophage numbers increased, the proportions of pro‐inflammatory subsets (iNOS^+^/CD68^+^, CD86^+^/CD68^+^) were significantly lower in the PEG/RGD‐PSLs@HAMA (36.00% ± 9.19%, 20.50% ± 2.95%) group compared to the sham (78.00% ± 4.60%, 72.83% ± 5.19%) and blank HAMA groups (50.83% ± 5.34%, 42.00% ± 6.19%). Conversely, the ratios of pro‐reparative subsets (Arg‐1^+^/CD68^+^, CD206^+^/CD68^+^) were already significantly higher in the treatment group at this early time point (Sham: 14.83% ± 2.48%, 21.67% ± 4.80%; HAMA: 28% ± 4.24%, 15.83% ± 1.94%; PEG/RGD‐PSLs@HAMA: 50.83% ± 5.34%, 42% ± 6.19%) (Figure 9A).

FIGURE 9The bioactive hydrogel modulates the wound immune microenvironment toward a pro‐reparative state. Immunohistochemical (IHC) analysis of macrophage phenotypes in wound tissues on day 3 (A) and day 7 (B). Right panels: Representative IHC staining for total macrophages (CD68) and polarization markers (iNOS and CD86 for M1; Arg‑1 and CD206 for M2). Black arrows: CD68^+^ macrophages; blue arrows: iNOS^+^ M1 macrophages; green arrows: CD86^+^ M1 macrophages; orange arrows: Arg‑1^+^ M2 macrophages; purple arrows: CD206^+^ M2 macrophages. Scale bars: 50 µm. Bottom panels: Quantitative analysis of macrophage subsets, expressed as the percentage of M1 (iNOS^+^ or CD86^+^) or M2 (Arg‑1^+^ or CD206^+^) cells among total CD68^+^ macrophages. Data are mean ± SD (*n* = 6); *p < 0.05, **p < 0.01, ***p < 0.001.

**Figure:**
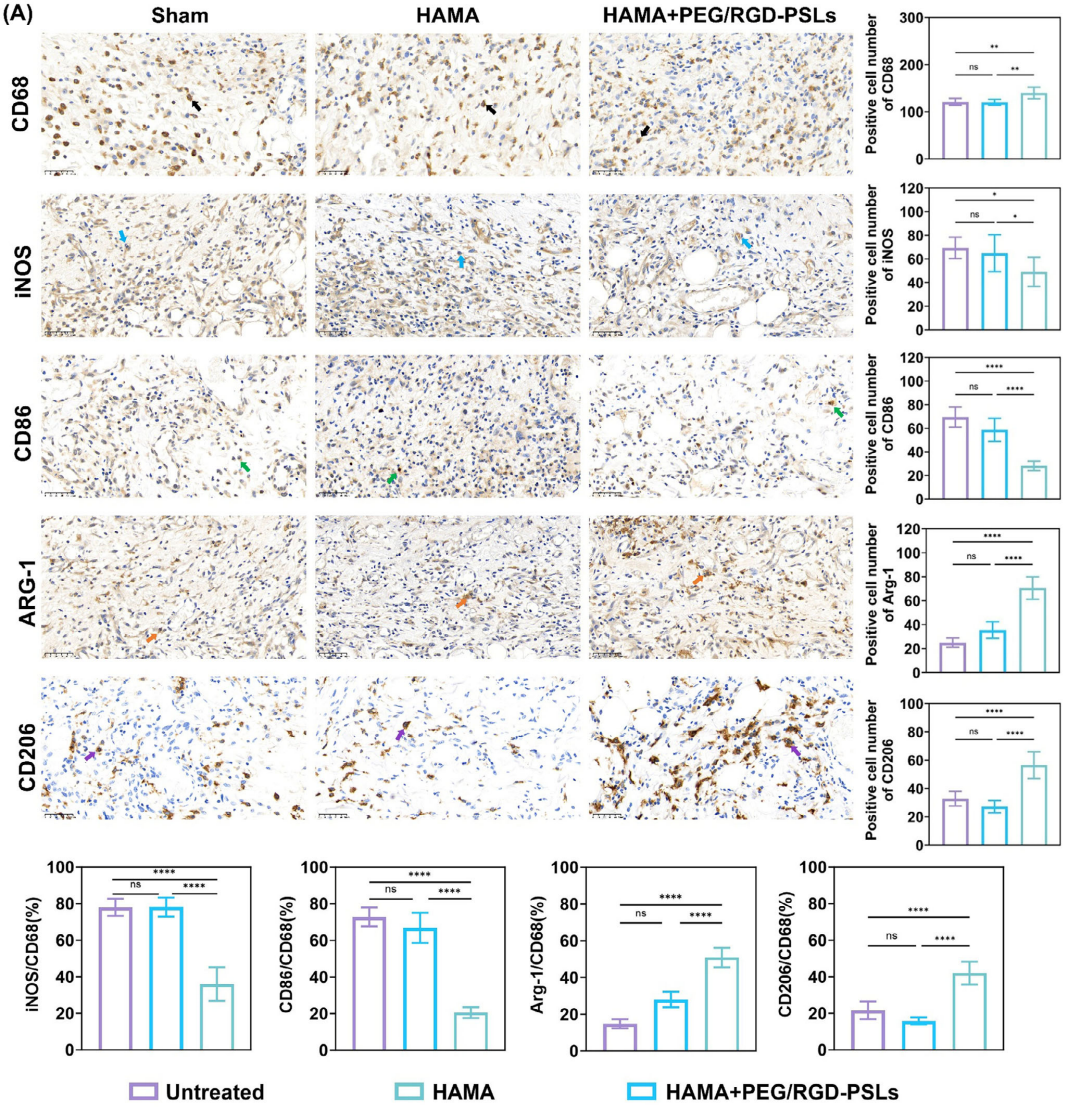

**Figure:**
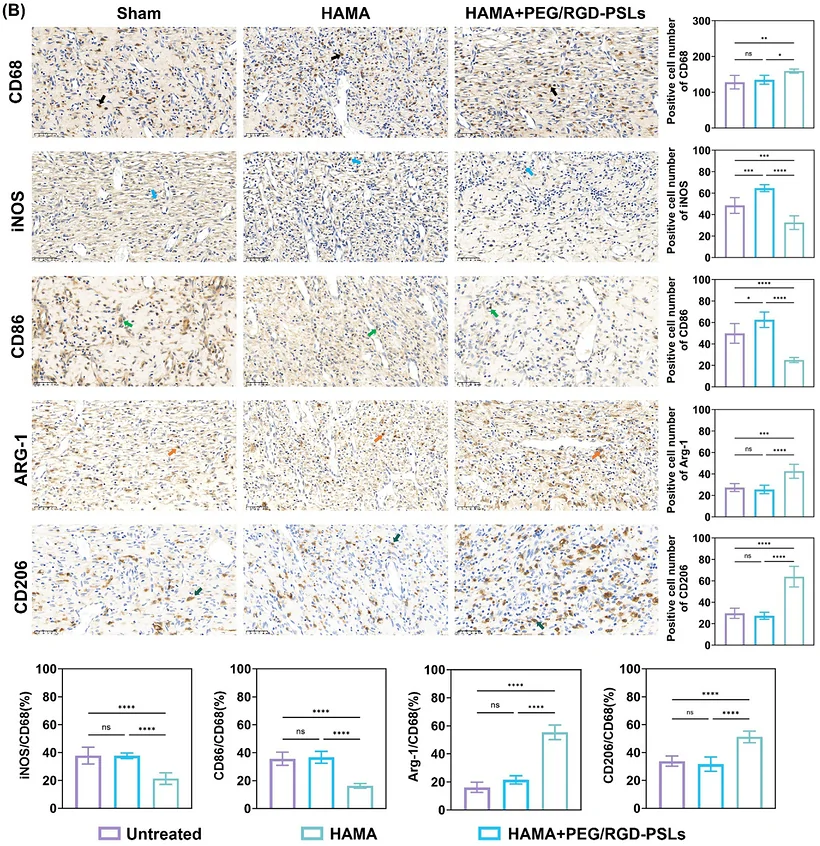

By day 7, this immunomodulatory shift became more pronounced. The proportions of iNOS^+^ and CD86^+^ M1‐associated macrophage subsets markedly decreased across all groups compared to day 3, yet remained lowest in the PEG/RGD‐PSLs@HAMA group. In striking contrast, the proportions of Arg‐1^+^ and CD206^+^ M2‐associated macrophage subsets increased further (55.33% ± 5.20%, 51.17% ± 4.17%), and was significantly higher in the bioactive hydrogel‐treated wounds than in the controls (Sham: 16.12% ± 3.66%, 33.83% ± 3.66%; HAMA: 21.5% ± 2.95%, 31.67% ± 5.13%) (Figure 9B). The analysis of cellular proportions confirmed this trend, with the M2/M1 phenotype ratio being highest in the treatment group.

These results clearly demonstrate that the PEG/RGD‐PSLs@HAMA hydrogel actively orchestrates macrophage behavior beyond mere recruitment. It promotes the migration of macrophages to the wound site while simultaneously and actively skewing their polarization toward the pro‐regenerative M2 phenotype, rather than exacerbating inflammation. This highlights the capacity of this bioactive material to function as an instructive immunomodulatory platform. The dynamic shift from a pro‐inflammatory to a pro‐reparative immune milieu in vivo robustly confirms the immunomodulatory effect observed in vitro and underscores its central role in facilitating organized diabetic wound healing.

## 3 Discussion

The nonhealing nature of diabetic wounds is fundamentally rooted in a self‐perpetuating cycle of chronic inflammation and cellular dysfunction. Current clinical management, while providing essential support, often fails to interrupt this pathological circuit [5, 9]. Our work presents a biomaterial strategy that transcends passive wound coverage by creating an active, instructive interface designed to directly reprogram the dysfunctional immune cells central to the pathology. By incorporating multifunctional PEG/RGD‐PSLs into a HAMA hydrogel, we engineered a localized therapeutic platform that simultaneously targets multiple facets of diabetic macrophage dysfunction.

The design is biomimetic and functionally layered. Surface‐exposed PS exploits the evolutionarily conserved “eat‐me” signal of physiological efferocytosis to engage immunomodulatory pathways. The conjugated RGD peptide provides targeting specificity to αvβ3 integrins overexpressed on activated wound macrophages, ensuring cellular specificity in a complex milieu [33, 34]. The strategic incorporation of PEG onto liposomes represents a critical design parameter that balances two opposing demands: initial “stealth” to avoid rapid clearance versus sustained receptor engagement to drive durable transcriptional reprogramming. Recent studies have highlighted that PEG density profoundly alters macrophage recognition and intracellular signaling dynamics [40, 41]. Our data demonstrate an optimal PEG molar ratio of 0.5 mol%: higher ratios (≥ 0.75 mol%) reduced cellular uptake and diminished anti‑inflammatory efficacy, whereas lower ratios (0.25 mol%) failed to sustain CD300a signaling beyond 12 h. This non‑monotonic behavior supports a model in which intermediate PEGylation provides sufficient steric stabilization to delay lysosomal degradation while still permitting receptor engagement via exposed RGD/PS motifs. At excessively high PEG densities, the dense “PEG brush” layer may physically hinder PS‑CD300a interactions, reducing signaling potency. Thus, our findings align with the emerging consensus that PEGylation must be carefully titrated to achieve prolonged immunomodulation without immune masking [41].

Our most pivotal mechanistic discovery is the delineation of the PS/CD300a/NF‐κB axis as the central pathway orchestrating macrophage reprogramming. Although CD300a is a recognized PS receptor known to transduce inhibitory signals that suppress LPS‐induced inflammation in other immune cells [39, 42], its role in diabetic macrophage dysfunction remained unclear. Herein, we identified a specific downregulation of CD300a on macrophages under diabetic conditions, revealing a novel defect contributing to the non‐resolving inflammation in chronic wounds. Crucially, treatment with PEG/RGD‐PSLs not only engaged this receptor but also actively reversed its deficit, restoring CD300a expression. The subsequent PS‐CD300a engagement transduced an intracellular inhibitory signal that directly attenuated the activity of the master inflammatory regulator, the MyD88/NF‐κB pathway [43, 44], leading to a broad downregulation of key inflammatory mediators (e.g., TNF‐α, IL‐6, IL‐1β, iNOS). This finding demonstrates a strategy to bypass the global impairment of efferocytosis in diabetes by precisely rescuing a specific receptor checkpoint, thereby reactivating a powerful intrinsic resolution circuit to silence pathological NF‐κB signaling.

To determine whether other PS‐binding receptors contribute to these anti‐inflammatory effects, we systematically analyzed expression changes of all known PS receptors using our RNA‑seq dataset. Treatment with PEG/RGD‐PSLs upregulated several such genes, including Trem2 (log_2_FC = 3.07, adj. *p* < 0.05) and Mfge8 (log_2_FC = 1.03, adj. *p* < 0.05), while downregulating Marcks, Thbs1, Havcr1, and Syt7. However, RT‑qPCR validation (Figure S4) revealed that among these, only Cd300a showed statistically significant upregulation upon treatment. Although other PS receptors can mediate efferocytosis and anti‑inflammatory effects in other contexts [27, 39], their lack of significant regulation in our system argues against a major functional contribution under diabetic‑mimicking conditions. This conclusion is further supported by siRNA knockdown experiments (Figure 7A–F), where CD300a silencing alone almost completely abolished the anti‑inflammatory effects of PEG/RGD‐PSLs.

Importantly, the dominance of CD300a is not a mere secondary consequence of global PS signaling. To distinguish receptor‑specific effects from a general response to PS engagement, we performed PPI and pathway correlation analyses (Figure 6D,G), which identified CD300a as a central node negatively correlated with NF‑κB and inflammatory pathways. The siRNA experiments provided the critical proof: depleting CD300a alone abrogated the anti‑inflammatory phenotype. If CD300a upregulation were simply a downstream byproduct of widespread PS signaling, its knockdown would not fully reverse the effect. Collectively, these data identify CD300a as the dominant and most robustly responsive PS receptor in this biomimetic platform, indicating that the biomimetic PS signal primarily exploits CD300a to reprogram macrophage inflammatory status under diabetic conditions.

Beyond immunomodulation, the platform conferred direct cytoprotection. It preserved mitochondrial ultrastructure, reduced intracellular and mitochondrial ROS (mtROS), and prevented pathological vesiculation under diabetic stress conditions. This ensures that reprogrammed macrophages are not merely phenotypically switched but are also metabolically competent and resilient, capable of executing sustained reparative functions in the hostile diabetic wound environment. This comprehensive functional rescue was directly translated into enhanced tissue‐level repair. The reprogrammed macrophages exhibited enhanced pro‐healing paracrine activity, accelerating fibroblast migration and, importantly, reducing oxidative stress in these stromal cells. In vivo, this manifested as accelerated wound closure characterized by high‐quality regeneration, featuring robust angiogenesis and organized collagen deposition. Critically, this regenerative outcome was preceded by a fundamental reprogramming of the wound immune microenvironment. The hydrogel first enhanced macrophage recruitment to the wound site, addressing a key deficit in diabetic healing [1, 12], and subsequently promoted a phenotypic shift from pro‐inflammatory M1 to pro‐reparative M2 polarization. This early and sustained transition toward an M2‐dominant environment is pivotal, as timely macrophage reprogramming is essential for resolving inflammation and initiating the proliferative phase of tissue repair [10, 11, 45].

The finding that diabetes selectively downregulates CD300a on wound macrophages, a defect rescued by exogenous PS signaling, adds to a growing paradigm in which chronic inflammatory diseases are associated with specific deficits in PS receptor expression or function. In aging, for instance, hyperactive p38 MAPK downregulates TIM‑4, impairing efferocytosis and prolonging inflammation, and local p38 inhibition has been proposed as a novel therapy for age‑related healing defects [25]. In cardiac repair, Mertk deficiency leaves apoptotic cardiomyocytes uncleared, exacerbating infarct necrosis and directly connecting efferocytic failure to organ dysfunction [24]. Unlike these examples—where receptor loss is driven by signaling dysregulation or genetic deficiency—the diabetic milieu suppresses Cd300a transcription, likely through hyperglycemia‑induced epigenetic mechanisms (e.g., DNA methylation, histone modifications, or miRNA alterations) that broadly affect gene expression in diabetic complications [45, 46]. Our siRNA experiments provide direct causal evidence that CD300a is both necessary and dominant for the anti‑inflammatory effects of PEG/RGD‑PSLs. Mechanistically, PS‑CD300a engagement recruits SHP‑1/SHP‑2 phosphatases to terminate NF‑κB activation [42]; thus, restoring CD300a expression may re‑establish a broken negative feedback loop. Although direct proof linking Cd300a repression to specific epigenetic marks is lacking, the well‑documented impact of sustained hyperglycemia on the epigenome supports this hypothesis and points to the CD300a/NF‑κB node as a uniquely vulnerable therapeutic target in diabetes.

Compared to existing clinical modalities (e.g., growth factors, advanced dressings, negative pressure therapy), our PS‑liposome strategy directly reinstates endogenous pro‑resolving signaling rather than indirectly promoting granulation or debridement, and avoids the hyperproliferative risks associated with growth factors. Clinical translation faces barriers including GMP‑compliant scalability, regulatory qualification as a biologic/combination product, and RGD synthesis costs, though targeted delivery may offset expenses and approved PEGylated liposomal drugs provide precedent. Biocompatibility is supported by clinically approved lipids and natural PS, with degradation products cleared via endogenous pathways. Future applications include topical/injectable formulations for diabetic foot ulcers, with patient stratification based on wound CD300a expression to optimize response.

While promising, several limitations warrant acknowledgment. First, although the STZ‐induced diabetic rat model is a well‑established platform for studying impaired wound healing, it induces type 1 diabetes via pancreatic β‑cell ablation, whereas human diabetic foot ulcers predominantly occur in the context of type 2 diabetes, which is characterized by insulin resistance, hyperinsulinemia, and metabolic syndrome components [47]. Second, the rat full‑thickness excisional model heals predominantly by wound contraction, whereas human wound healing relies more on re‑epithelialization and granulation tissue formation; splinting rings were not used in this study [48, 49]. Future studies will address these limitations by employing more clinically relevant models, including high‑fat diet/low‑dose STZ‑induced type 2 diabetes models and db/db mice, as well as splinted rats or porcine models to better mimic human healing. Moreover, validation in human primary diabetic wound macrophages will be essential to strengthen translational relevance.

In summary, we have developed an immunoregenerative hydrogel that directly counteracts the core pathophysiological mechanisms of diabetic wounds. By delivering a biomimetic PS signal to selectively modulate the CD300a/NF‐κB axis, the hydrogel concurrently alleviates chronic inflammation, confers metabolic cytoprotection, and promotes a regenerative wound microenvironment. This work establishes a systems‐level therapeutic strategy with translational potential for diabetic foot ulcers and other chronic inflammatory disorders.

## 4 Conclusion

In conclusion, we have developed an immunomodulatory hydrogel that actively reverses the core pathological mechanisms of diabetic wound healing. By integrating biomimetic, multifunctional PSLs into a photocrosslinkable hydrogel, we created a sustained‐release platform that targets dysfunctional macrophages. The hydrogel promotes recruitment, drives M1‐to‐M2 repolarization, and provides cytoprotection. The pivotal mechanism involves rescuing the expression and function of the inhibitory PS receptor CD300a, leading to suppression of the pro‐inflammatory MyD88/NF‐κB pathway. This concerted action resulted in accelerated wound closure and improved healing quality in a diabetic rat model. This work establishes a new paradigm for engineering intelligent, system‐resetting biomaterials to treat chronic, inflammation‐driven tissue repair disorders.

## 5 Materials and Methods

### 5.1 Materials and Reagents

Hyaluronic acid (HA), and lithium phenyl‐2,4,6‐trimethylbenzoylphosphinate (LAP) were purchased from Sigma–Aldrich. L‐α‐PS (porcine brain), L‐α‐phosphatidylcholine (PC, egg yolk), cholesterol (CH), 1,2‐distearoyl‐sn‐glycero‐3‐phosphoethanolamine‐N‐[methoxy(polyethylene glycol)‐2000] (ammonium salt) (18:0 PEG2000 PE), and 1,2‐dioleoyl‐sn‐glycero‐3‐phosphoethanolamine‐N‐[4‐(p‐maleimidomethyl) cyclohexane‐carboxamide] (DSPE‐RGD) were obtained from Avanti Polar Lipids (Alabaster, AL, USA). All other reagents were purchased from Sigma–Aldrich unless otherwise specified.

### 5.2 Synthesis and Characterization of PEG/RGD‐PSLs

PEG/RGD‐PSLs were synthesized using the well‐established thin‐film hydration and extrusion method [30]. Briefly, lipids, including L‐α‐PS, L‐α‐phosphatidylcholine (PC), cholesterol (CH), DSPE‐RGD, and PEG2000 PE (for PEGylated formulations), were dissolved in a chloroform/methanol mixture (9:1, *v*/*v*) at the specified molar ratios. For the standard RGD‐PSLs, the lipid composition was fixed at PS: PC:CH:DSPE‐RGD = 50:22:25:3 (molar ratio). To prepare PEG/RGD‐PSLs, PC was partially substituted with PEG2000 PE at 0.25, 0.5, 0.75, and 1.0 mol% of the total lipids. Phosphatidylcholine liposomes (PCLs), used as a non‐PS control, were prepared by substituting PS with an equimolar amount of PC while keeping other procedures identical. Briefly, the organic solvent was gently evaporated under a stream of nitrogen to form a thin, uniform lipid film on the inner wall of a glass vial. The film was further desiccated under vacuum for at least 2 h to remove any trace organic solvent. Subsequently, the dried lipid film was hydrated with phosphate‐buffered saline (PBS, pH 7.4) for 1 h with intermittent vortexing to form multilamellar vesicles (MLVs). The resulting MLV suspension was sequentially extruded through polycarbonate membranes with pore sizes of 400 and 100 nm using a mini‐extruder (Avanti Polar Lipids) to obtain homogeneous vesicles.

The morphology and size of the prepared liposomes were examined by TEM (Cryo‐TEM, ThermoFisher Helios G4 UC). The hydrodynamic diameter and zeta potential were determined by DLS using a particle analyzer (Anton Paar, Litesizer 500). Successful conjugation of PEG to the liposome surface was confirmed by ^1^H Nuclear Magnetic Resonance (^1^H‐NMR) spectroscopy (NMR, Agilent 800 MHz).

### 5.3 Synthesis of HAMA and Fabrication of Bioactive Hydrogel

HAMA was synthesized in accordance with the method reported by Burdick et al. [50]. Briefly, HA was reacted with methacrylic anhydride in ice‐cold PBS (pH 8.0) for 24 h, purified by dialysis, and lyophilized. HA methacrylation was determined by ^1^H Nuclear Magnetic Resonance (^1^H‐NMR, AVANCE IIITM HD 600 MHz). FT‐IR spectra were recorded using a Nicolet 6700 FT‐IR spectrometer (Thermo Scientific) equipped with an attenuated total reflection (ATR) accessory.

For hydrogel fabrication, the precursor solution was prepared by mixing 1 mL of HAMA (20% *w*/*v*), 1 mL of PEG/RGD‐PSLs (0.34 mg/mL lipid concentration), and 2 mL of PBS containing 0.5% *w*/*v* LAP. This mixture was then photocrosslinked under UV light (405 nm) for 60 s to form the covalently crosslinked network. To characterize the incorporation of liposomes within the hydrogel matrix, hydrogels were frozen in liquid nitrogen and subsequently lyophilized. The cross‐sectional morphologies were examined by sputter‐coating with gold and imaging using a field emission scanning electron microscope (Sigma 300, Zeiss). The photopolymerization kinetics and viscoelastic properties of hydrogels were analyzed using a HAAKE MARS III rheometer. For degradation studies, pre‐weighed hydrogels were incubated in PBS at 37°C. At specified intervals, samples were removed, washed, lyophilized, and weighed. The remaining mass percentage was calculated relative to the initial dry weight. Degradation was monitored by weight loss over 21 days.

### 5.4 In Vitro Biological Evaluation

#### 5.4.1 Cell Culture and Treatments

RAW 264.7 murine macrophages and NIH 3T3 cells were cultured in DMEM supplemented with 10% FBS at 37°C in 5% CO_2_. Diabetic conditions were simulated using high glucose (25 mM) DMEM with lipopolysaccharide (LPS) stimulation at 200 ng/mL.

#### 5.4.2 In Vitro Cellular Uptake of Liposomes

To investigate the role of PEGylation and RGD/PS in modulating the macrophage uptake of liposomes, a comparative study was conducted. Liposomes were fluorescently labeled with 0.5 mol% N‐(tetramethylrhodamine‐6‐thiocarbamoyl)‐1,2‐dihexadecanoyl‐sn‐glycero‐3‐phosphoethanolamine (TRITC‐DHPE; Biotium Inc., Hayward, CA, USA). RAW 264.7 were cultured with fluorescently labeled liposomes in the presence of LPS (Escherichia coli, serotype 055: B5) at 200 ng/mL to mimic an inflammatory (M1‐like) state. At predetermined time points (1, 3, 6, 12, and 24 h), the fluorescence intensity of cells was analyzed using a flow cytometer (Sony SA380), with 10 000 events recorded per sample. For qualitative visualization and intracellular localization, macrophages grown on a confocal dish were treated as above. After 3 and 12 h of incubation with liposomes, cells were washed, and the plasma membrane was stained with DiOC_18_ (3) (3,3′‐dioctadecyloxacarbocyanine perchlorate, 5 µM) for 10 min at 37°C. Cell images were acquired using a confocal laser scanning microscope (NIKON Ti).

#### 5.4.3 Migration Assays

Cell migration was assessed using a Transwell chamber system with 8 µm pore polycarbonate membranes. To assess the specific effects of liposomes on the migration of polarized macrophage subtypes, macrophages seeded in the upper chamber were pretreated with either IL‐4 (20 ng/mL) or LPS (200 ng/mL) for 24 h to induce M2 or M1 phenotypes, respectively. Subsequently, the corresponding liposome formulations, suspended in fresh serum‐free medium, were added to the lower chamber. Following a 12‐h incubation period, migrated cells on the lower surface of the membrane were fixed with 4% paraformaldehyde, stained with 0.1% crystal violet, imaged under a light microscope, and quantified by counting cells across three randomly selected microscopic fields per membrane.

#### 5.4.4 Macrophage Polarization and Reprogramming Assays

Macrophage polarization was assessed by gene expression analysis and surface marker detection. Cells were seeded in 12‐well plates at 1.5 × 10^5^ cells per well. To evaluate the capacity of liposomes to suppress classical M1 polarization, macrophages were co‐treated with LPS (200 ng/mL) and the respective liposome formulations for 3, 6, 12, and 24 h. Total RNA was extracted using TRIzol reagent. cDNA was synthesized with a reverse transcription kit. The mRNA expression levels of pro‐inflammatory genes (IL‐1β, IL‐6, TNF‐α, and iNOS) were quantified by real‐time PCR using ViiA V7Dx. Gene expression was normalized to the housekeeping gene GAPDH. The expression of inducible nitric oxide synthase (iNOS), a canonical M1 marker, was analyzed by confocal microscopy and flow cytometry after 12 h of treatment. Cells were fixed, permeabilized, blocked with 3% BSA, and incubated with an APC‐conjugated anti‐iNOS antibody (1:200 dilution; BioLegend, San Diego, CA, USA) for 1 h at room temperature. For confocal imaging (NIKON Ti), nuclei were counterstained with DAPI. For flow cytometry (Sony SA380), 10 000 single‐cell events were recorded, and the mean fluorescence intensity of the APC channel was analyzed.

To evaluate the capacity of liposomes to reprogram M1‐polarized macrophages toward an M2 phenotype, macrophages were first polarized to an M1 state by stimulation with LPS (200 ng/mL) for 12 h. The medium was then replaced with fresh medium containing the respective liposome formulations (without LPS) for a further 12‐h incubation. The mRNA expression levels of characteristic M2‐associated genes (Arg‐1, FIZZ‐1 (found in the inflammatory zone), CD206, and YM‐1 (chitinase‐like protein 3)) were analyzed as described above. The expression of Arginase‐1 (Arg‐1) protein was detected by staining with an APC‐conjugated anti‐Arg‐1 antibody (1:200; BioLegend) followed by confocal and flow cytometry.

#### 5.4.5 Intracellular ROS and Mitochondrial Function Assessment

Intracellular total ROS levels were measured using the fluorescent probe DCFH‐DA. Treated macrophages were incubated with 10 µM DCFH‐DA for 30 min at 37°C, washed, and fluorescence intensity was quantified by flow cytometry. For mitochondrial analysis, cells were co‐stained with 200 nM MitoTracker Green FM (30 min) and 5 µM MitoSOX Red (15 min). Fluorescence intensity was quantified by flow cytometry. Mitochondrial morphology was further examined by TEM as described in Section 5.4.6.

#### 5.4.6 Ultrastructural Analysis by Electron Microscopy

The ultrastructural morphology of macrophages under diabetic‐mimicking stress and following treatment was examined using scanning (SEM, JSM‐IT700HR InTouchSoope) and TEM (JEM‐1400FLASH) to visualize both surface alterations and intracellular organelle integrity. Images were captured to assess specific ultrastructural features, including mitochondrial morphology (cristae structure, swelling), endoplasmic reticulum integrity, and the presence and morphology of lysosomes.

#### 5.4.7 Macrophage‐Fibroblast Co‐Culture Scratch Assay and Fibroblast ROS Detection

To evaluate the functional contribution of macrophage reprogramming to fibroblast‐mediated wound closure in vitro, a scratch wound assay was performed using a transwell co‐culture system. RAW 264.7 macrophages in the upper chamber of a 6‐well plate were stimulated with LPS (200 ng/mL, 12 h) to induce M1 polarization, followed by treatment with liposome formulations (RGD‐PSLs or PEG/RGD‐PSL) in serum‐free medium for an additional 12 h. Concurrently, NIH 3T3 fibroblasts were seeded at 2 × 10^5^ cells per well and cultured to confluence. After macrophage conditioning, the fibroblast monolayer was wounded uniformly using a sterile 200 µL pipette tip. Inserts containing conditioned macrophages were then transferred into the lower wells to establish a co‐culture system in serum‐free DMEM. Wound images were acquired immediately (0 h) and at 12, 18, and 24 h using a digital microscope. Wound areas were measured using ImageJ software, and the percentage of wound closure was calculated to assess migration and repair dynamics over time. In a parallel setup, intracellular ROS levels were measured using the fluorescent probe DCFH‐DA. Briefly, after 12 h of co‐culture, NIH 3T3 cells were then assessed for intracellular ROS levels using the DCFH‐DA assay as described in Section 5.4.5.

#### 5.4.8 RNA Sequencing and Omics Analysis

Total RNA was extracted from macrophages under diabetic‐mimicking conditions following 12‐h stimulation with (1) HG: high glucose (25 mM D‐glucose), (2) HG + LPS: high glucose (25 mM) + lipopolysaccharide (200 ng/mL), and (3) HG + LPS + PEG/RGD‐PSLs, using TRIzol reagent (Invitrogen) and mechanical homogenization (30 Hz, 2 min). After chloroform‐based phase separation, RNA was precipitated with isopropanol (−20°C, overnight), washed with 75% DEPC‐treated ethanol, and resuspended in 30 µL DEPC‐water. RNA quality was confirmed by NanoDrop 2000 (A260/A280 ratio: 1.8–2.1) and Agilent 2100 Bioanalyzer (RIN > 8.0). For RNA‐seq, 1 µg of total RNA was used to construct cDNA libraries using the NEBNext Ultra II Library Prep Kit (NEB): mRNA was enriched via oligo(dT) beads, fragmented to ∼200 bp, followed by first‐strand synthesis, second‐strand synthesis, end repair, adaptor ligation, and 12‐cycle PCR amplification. Final libraries (250–300 bp) were validated using the Bioanalyzer and qPCR. Sequencing was performed on an Illumina NovaSeq 6000 platform (BGI) with paired‐end 150 bp reads, yielding approximately 6 Gb per sample. Raw reads were assessed using FastQC (v0.11.9) and filtered with Trimmomatic (v0.39) to remove adapters, low‐quality bases (Phred score < 20), and reads containing more than 10% N bases. Clean reads were aligned to the mouse reference genome (GRCm38) using HISAT2 (v2.2.1), and read counts were generated using featureCounts (v2.0.3) based on the corresponding GENCODE mouse annotation. Differentially expressed genes (DEGs) were identified using DESeq2 (v1.34.0) in R (v4.1.2) with an adjusted *p* value < 0.05 and |log2 fold change| > 1 as thresholds. Functional enrichment analysis of GO terms and KEGG pathways was conducted using clusterProfiler (v4.2.2) with statistical significance set at adjusted *p *< 0.05. PPI networks, heatmaps, volcano plots, and GSEA were visualized using STRING (v11.5), Gephi (v0.9.2), TBtools (v1.09876), GraphPad Prism (v9.3.1), and OmicShare tools.

#### 5.4.9 Mechanistic Protein Analysis

Protein lysates from treated macrophages were subjected to Western blotting. Primary antibodies against CD300a, MYD88, phospho‐MYD88, phospho‐NF‐κB p65, total NF‐κB p65, and β‐actin (loading control) were used according to manufacturers' protocols. Band intensities were quantified using ImageJ software.

#### 5.4.10 siRNA‑Mediated CD300a Knockdown

RAW 264.7 macrophages were seeded in 6‑well plates at a density of 2 × 10^5^ cells/well and cultured overnight. Two independent siRNA sequences targeting mouse Cd300a were used: si‑CD300a‑188 (sense: 5′‑CACUUAAGAUACUAUGUAATT‑3′, antisense: 5′‑UUACAUAGUAUCUUAAGUGTT‑3′), and si‑CD300a‑756 (sense: 5′‑GCUACCAAGUCAGGUAGAATT‑3′, antisense: 5′‑UUCUACCUGACUUGGUAGCTT‑3′). A negative control siRNA (si‑NC: sense: 5′‑UUCUCCGAACGUGUCACGUTT‑3′, antisense: 5′‑ACGUGACACGUUCGGAGAATT‑3′) was used as control. Transfection was performed using Lipofectamine 3000 (Invitrogen) at a final siRNA concentration of 50 nM, following the manufacturer's protocol. After 24 h of transfection, cells were incubated under diabetic‑mimicking conditions (high glucose, 25 mM) with LPS (200 ng/mL) in the presence or absence of PEG/RGD‑PSLs for an additional 3 or 12 h (for Western blot) and 12 h (for RT‑qPCR). Knockdown efficiency was confirmed by RT‑qPCR. Both si‑CD300a‑188 and si‑CD300a‑756 were used for RT‑qPCR analyses, whereas si‑CD300a‑756 was carried forward for Western blot validation.

### 5.5 In Vivo Diabetic Wound Healing Model

#### 5.5.1 Animal Model and Wound Creation

All animal procedures were conducted in strict accordance with protocols approved by the Science and Technology Ethics Committee of Nanjing University (IACUC‐D2304018). Male Sprague–Dawley rats (8 weeks old) were housed under standard conditions. Diabetes was induced via five consecutive daily intraperitoneal injections of streptozotocin (STZ, 50 mg/kg body weight, dissolved in 0.1 M citrate buffer, pH 4.5). Rats with persistent non‐fasting blood glucose levels > 16.7 mmol/L, measured using a glucometer over two consecutive weeks, were used for wounding studies. Full‐thickness excisional wounds (8 mm diameter) were created on the shaved and disinfected dorsal skin of anesthetized rats using a sterile biopsy punch. Diabetic rats were randomly assigned to the following four groups (*n* = 6 rats per group): (1) Sham Group: Wounds were left uncovered as a non‐treated control. (2) Hydrogel Group: Wounds were covered with 50 µL of sterile HAMA hydrogel. (3) PEG/RGD‐PCLs@HAMA Group: Wounds were covered with 50 µL of HAMA hydrogel containing PEG/RGD‐PCLs. (4) PEG/RGD‐PSLs@HAMA Group: Wounds were covered with 50 µL of the active therapeutic hydrogel containing PEG/RGD‐PSLs. All hydrogels were applied immediately post‐wounding and crosslinked in situ via exposure to UV light for 60 s. Wound areas were photographed on days 0, 3, 7, 10, and 14 with a metric scale. The wound area was quantified using ImageJ software, and the percentage of wound closure was calculated. Wound tissues were harvested at predetermined endpoints on days 3, 7, and 14 post‐injury and processed for histology. Histological analyses shown in Figures 8B–E and 9 were performed for the Sham, HAMA, and PEG/RGD‐PSLs@HAMA groups.

#### 5.5.2 Histological and IHC Analysis

Harvested wound tissues were fixed in 4% paraformaldehyde, paraffin‐embedded, and sectioned at 5 µm thickness. Sections were stained with hematoxylin and eosin (H and E) staining for general morphological assessment. Collagen deposition and organization were assessed by Masson's trichrome staining. For cellular phenotyping, antigen retrieval was performed. After blocking, sections were incubated overnight at 4°C with primary antibodies against CD68 (macrophages), Inos, and CD86 (M1 markers), and Arg‐1 and CD206 (M2 markers). Following incubation with appropriate biotinylated secondary antibodies and HRP‐conjugated streptavidin, signals were developed using a DAB substrate kit and counterstained with hematoxylin. Slides were imaged with a bright‐field microscope, and positive cells were quantified in five random high‐power fields per sample using ImageJ.

### 5.6 Statistical Analysis

Data are presented as mean ± SD. Comparisons among multiple groups at a single time point were performed by one‐way ANOVA with Tukey's post‐hoc test. Time‐course/group‐by‐time data were analyzed by two‐way ANOVA with Tukey's multiple‐comparisons test, and longitudinal wound‐closure data were analyzed by two‐way repeated‐measures ANOVA with Tukey's multiple‐comparisons test using GraphPad Prism 9. *p* < 0.05 was considered significant.

## Author Contributions

Authorship order among the co‐first authors was determined according to their relative contributions. The specific contributions are as follows: Conceptualization: **Xin Tong**, **Lele Wu**, and **Mengmeng Liu**. Methodology: Lele Wu, **Xuetong Hao**, **Yang Zou**, **Xinglin Chen**, and **Junyan Kong**. Investigation: Lele Wu (lead, hydrogel fabrication, liposome synthesis, in vitro studies), Xuetong Hao (lead, animal experiments, histology), Yang Zou (lead, mechanistic studies), Xinglin Chen, Junyan Kong, **Chunning Gu**, and **Wenjie Ma**. Formal analysis: Yang Zou, Lele Wu, Xuetong Hao, and Mengmeng Liu. Data curation: Lele Wu, Xuetong Hao, and Yang Zou. Software: Xuetong Hao. Validation: Xinglin Chen and Chunning Gu. Resources: Chunning Gu and Wenjie Ma. Writing – original draft: Lele Wu, Xuetong Hao, Yang Zou. Writing – review and editing: Xin Tong, Mengmeng Liu, **Huifen Liu**, and all authors. Visualization: Yang Zou, Lele Wu, Xuetong Hao, and **Zhongjun Yang**. Supervision: Xin Tong, Mengmeng Liu, Huifen Liu, and Zhongjun Yang. Project administration: Xin Tong and Huifen Liu. Funding acquisition: Xin Tong, Lele Wu, and Mengmeng Liu.

## Funding

This work was supported by the National Natural Science Foundation of China (Grant number: 82201132), the Shandong Provincial Natural Science Foundation Excellent Youth Science (Overseas) Project (Grant number: 2025HWYQ‐018), and Nanjing Health Science and Technology Development Project (Grant number: YKK23178).

## Ethics Statement

All animal experiments were conducted in strict accordance with the National Institutes of Health (NIH) guide for the care and use of laboratory animals and were approved by the Science and Technology Ethics Committee of Nanjing University (Approval number: IACUC‐D2304018).

## Conflicts of Interest

The authors declare no conflicts of interest.

## Supporting information




**Supporting File**: adhm71648‐sup‐0001‐SuppMat.docx.

## Data Availability

All data associated with this study are available in the main text or Supporting Information. The RNA‐sequencing data are available from the corresponding author upon reasonable request.
