# Supplementary material for: Targeting CD300a Signaling With a Phosphatidylserine‐Presenting Hydrogel Rescues Macrophage Dysfunction and Accelerates Diabetic Wound Healing
Source: Adv Healthc Mater. 2026 Aug 30;15(40):e71648. doi: 10.1002/adhm.71648 (PMC13712216; doi:10.1002/adhm.71648)
Supplement: Supplementary file 1 [file ADHM-15-0-s001.docx]

Supporting Information

**Targeting CD300a Signaling with a Phosphatidylserine-Presenting Hydrogel Rescues Macrophage Dysfunction and Accelerates Diabetic Wound Healing**

Lele Wu^1#^, Xuetong Hao^1#^, Yang Zou^1#^, Xinglin Chen^1^, Junyan Kong^1^, Chunning Gu^1^, Wenjie Ma^1^, Zhongjun Yang^2^*, Huifen Liu^1^*, Mengmeng Liu^2^* and Xin Tong^1^*

**Supplementary Figure S1. (Related to Figure 1) Additional characterization data.**

(A) Hydrodynamic diameter distribution (by intensity) of RGD-PSLs and PEG/RGD-PSLs with increasing PEGylation ratios, as measured by dynamic light scattering (DLS). Corresponding zeta potential values of the liposome formulations, demonstrating a PEGylation-dependent reduction in surface charge. Data are presented as mean ± SD (n=3).

(B) Full ¹H NMR spectra of RGD-PSLs components.

**Supplementary Figure S2. (Related to Figure 3) Transcriptomic analysis reveals PEG/RGD-PSLs modulate oxidative stress response pathways.**

(A–D) Flow cytometry analysis of (A) intracellular total ROS, (B) MitoTracker Green, (C) mitochondrial ROS, and (D) fibroblast ROS after co-culture. Data are mean ± SD (n=4); **p* < 0.05, ***p* < 0.01, ****p* < 0.001. (E) Gene Ontology (GO) enrichment analysis (Biological Process) of genes differentially expressed after PEG/RGD-PSLs treatment under diabetic-mimicking (HG + LPS) conditions. The bubble chart shows the top enriched GO terms, with “cellular response to oxidative stress”. Bubble size represents gene count, and color denotes -log10(p-value). (F) Heatmap of differential expression patterns for a subset of key genes belonging to the “cellular response to oxidative stress” term, shown as log2 fold-change relative to respective controls (LPS vs. NC, PEG/RGD-PSLs vs. LPS).

**Supplementary Figure S3. (Related to Figure 4) Comprehensive transcriptomic analysis.** (A) Principal Component Analysis (PCA) plot of RNA-seq samples from different treatment groups. (B) Venn diagram showing the overlap of DEGs between the LPS vs. NC and LPS + PEG/RGD-PSLs vs. LPS comparisons. (C) Number of up- and down-regulated genes in the HG + LPS group versus normal control (NC). (D) Volcano plot of differentially expressed genes (DEGs) in macrophages treated with LPS versus the NC group under diabetic-mimicking conditions (HG + LPS). (E, F) KEGG pathway and GSEA analysis of pathways activated by HG + LPS stimulation. (G) KEGG pathway analysis showing regulation of metabolic pathways (oxidative phosphorylation, TCA cycle, pyruvate metabolism) after PEG/RGD-PSLs treatment. (H) Heatmap depicting the expression profile of TCA cycle and oxidative phosphorylation genes regulated by PEG/RGD-PSLs. (I) Number of genes significantly reversed (up or down) by PEG/RGD-PSLs treatment.

**Supplementary Figure S4.** RT‑qPCR analysis of CD300a, Mfge8, and Trem2 mRNA expression in each group. Data are mean ± SD (n = 3 independent experiments). **p* < 0.05, ***p* < 0.01, ****p* < 0.001; n.s., not significant.

**Supplementary Figure S5.** PEG/RGD‑PSLs@HAMA accelerates re‑epithelialization and promotes mature epidermal structure formation. Representative H&E‑stained wound sections at day 3, day 7 and day 14. Scale bars: 50 µm. Black arrows indicate the migrating epithelial structures.
